# MicroRNA-141 enhances anoikis resistance in metastatic progression of ovarian cancer through targeting KLF12/Sp1/survivin axis

**DOI:** 10.1186/s12943-017-0582-2

**Published:** 2017-01-17

**Authors:** Celia S. L. Mak, Mingo M. H. Yung, Lynn M. N. Hui, Leanne L. Leung, Rui Liang, Kangmei Chen, Stephanie S. Liu, Yiming Qin, Thomas H. Y. Leung, Kai-Fai Lee, Karen K. L. Chan, Hextan Y. S. Ngan, David W. Chan

**Affiliations:** 1 Department of Obstetrics and Gynaecology, L747 Laboratory Block, LKS Faculty of Medicine, The University of Hong Kong, 21 Sassoon Road, Hong Kong SAR, People’s Republic of China; 2 School of Biomedical Sciences, LKS Faculty of Medicine, The University of Hong Kong, 21 Sassoon Road, Hong Kong SAR, People’s Republic of China

**Keywords:** miR-141, Anoikis resistance, Ovarian cancer, KLF12, Sp1, survivin

## Abstract

**Background:**

Cancer metastasis is determined by the formation of the metastatic niche and the ability of cancer cells to adapt to microenvironmental stresses. Anoikis resistance is a fundamental feature of metastatic cancer cell survival during metastatic cancer progression. However, the mechanisms underlying anoikis resistance in ovarian cancer are still unclear.

**Methods:**

Expressions of miRNA-141 and its downstream targets were evaluated by qPCR, Western blotting, Immunohistochemical (IHC) and *in situ* hybridization (ISH) assays. The luciferase assays were used to prove KLF12 as the downstream target of miR-141. The cDNA microarray and apoptotic protein arrays were used to identify the targets of miR-141 and KLF12. The competition of KLF12 and Sp1 on survivin promoter was examined by ChIP assay. IHC analysis on ovarian cancer tissue array was used to evaluate the expressions of KLF12 and miR-141 and to show the clinical relevance. The functional studies were performed by in vitro and in vivo tumorigenic assays.

**Results:**

Enforced expression of miR-141 promotes, while knockdown of miR-141 expression inhibits, cell proliferation, anchorage-independent capacity, anoikis resistance, tumor growth and peritoneal metastases of ovarian cancer cells. Bioinformatics and functional analysis identified that Kruppel-related zinc finger protein AP-2rep (KLF12) is directly targeted by miR-141. Consistent with this finding, knockdown of KLF12 phenocopied the effects of miR-141 overexpression in ovarian cancer cells. In contrast, restoration of KLF12 in miR-141-expressing cells significantly attenuated anoikis resistance in ovarian cancer cells via interfering with Sp1-mediated survivin transcription, which inhibits the intrinsic apoptotic pathway and is crucial for ovarian cancer cell survival, anoikis resistance and peritoneal metastases. Immunohistochemical (IHC) and in situ hybridization (ISH) assays confirmed that miRNA-141 expression is inversely correlated with KLF12 expression and significantly associated with advanced ovarian cancers accompanied with distal metastases, underscoring the clinical relevance of our findings.

**Conclusions:**

Our data identify a novel signaling axis of miR-141/KLF12/Sp1/survivin in enhancing anoikis resistance and likely serves as a potential therapeutic target for metastatic ovarian cancer.

**Electronic supplementary material:**

The online version of this article (doi:10.1186/s12943-017-0582-2) contains supplementary material, which is available to authorized users.

## Background

Epithelial ovarian cancer (EOC) is the most lethal gynecological malignancy worldwide. This disease is generally called the “silent killer” because there are no symptoms, and thus, the majority of patients are diagnosed when the cancer is at an advanced stage with extensive micrometastases [1]. Most deaths from this cancer are attributed to metastatic progression [1]. Therefore, understanding the molecular mechanisms of metastases may assist in the development of targeted therapies to improve the outcomes of this disease. Cancer metastasis is determined by the formation of the metastatic niche and the ability of cancer cells to adapt to the microenvironment [2]. However, the associated molecular mechanisms remain unclear.

Metastasis is a multistep process that allows cancer cells to spread from the primary site to a distant location in the body and includes local invasion, intravasation, circulation, extravasation, proliferation and angiogenesis [3]. Among the steps that occur during cancer cell metastasis, escaping apoptosis (i.e., anoikis resistance) while detaching from the extracellular matrix or neighboring cells at the primary sites and circulating in transit to a distant location is of utmost importance. Many studies have suggested that the upregulation of oncogenes or the loss of tumor suppressors in the signaling pathways associated with the death receptor (extrinsic), mitochondrial (intrinsic) and convergence pathways (IAPs) allows tumor cells to circumvent anoikis to evade restriction by integrin–ECM interactions [4]. Unlike other cancers, peritoneal metastases is the most common route of ovarian cancer metastasis [5]. But similar to other cancers, micrometastases is the commonly cause of ovarian cancer recurrence as well as chemoresistance [6]. The detached ovarian cancer cells acquire anoikis resistance to survive in the ascitic fluid prior to metastatic colonization in the omentum/peritoneum is a vital trait of metastatic progression [7, 8].

Emerging evidence has indicated that deregulation of miRNAs is involved in cancer metastases [9]. MicroRNAs (miRNA) are small endogenous, non-coding RNAs of 21 to 25 nucleotides [10, 11] that negatively regulate gene expression by translational repression or endonucleolytic cleavage of the target mRNAs [12, 13] at the post-transcriptional level. Since 2002, emerging evidence has suggested that the aberrant expression of miRNAs in human cancers may modulate tumor progression by regulating tumor suppressors and oncogenes [14]. For example, miR-221/222 has been shown to increase chemoresistance in breast cancer [15], increase tumorigenicity of cancer cells through PTEN [16], promote human castration-resistant prostate cancer development [17] and stimulate invasion in glioblastoma [18]. Another example is the miR-200 family, which facilitates endometrioid carcinoma growth by modulating the Notch, p53, MAPK, and ErbB signaling pathways [19, 20]. However, the functional roles of miRNAs associated with anoikis resistance in human ovarian cancer have rarely been investigated.

In this study, we found that a member of the miR-200 family, miR-141, is prominently overexpressed in advanced and metastatic ovarian cancers. Using a series of functional and biochemical analyses, we identified that miR-141 enhances resistance against microenvironmental stresses and anoikis by targeting and repressing KLF12 expression, which allows Sp1 to upregulate survivin expression, an inhibitor of intrinsic apoptotic pathway, is crucial in promoting cell survival, proliferation and peritoneal metastases of ovarian cancer.

## Methods

### Cell culture and human tissue samples

The human ovarian cancer cell lines; SKOV3 (purchased from American Type Culture Collection, ATCC), OVCA433 and A2780cp and cervical cancer cell lines (OV2008 and C13*) (kindly provided by Prof. B. Tsang, Department of Obstetrics and Gynecology, University of Ottawa) were used in this study. They were grown either in Dulbecco’s modified Eagle’s medium (DMEM) or in minimum essential medium (MEM) (Invitrogen, Grand Island, N.Y., USA) supplemented with 10% fetal bovine serum (FBS) (Invitrogen) and 1% penicillin-streptomycin (P/S) (Invitrogen). Three human immortalized ovarian surface epithelial cell lines, HOSE 17–1, HOSE 11–12 and HOSE 96-9-10 (kindly provided by Prof. GSW. Tsao, The University of Hong Kong) [21], were cultured in Medium 199 and Medium 105 (Sigma-Aldrich Corp., St. Louis, MO, USA) supplemented with 10% FBS and 1% P/S. All cells were incubated in an incubator containing 5% CO_2_ at 37 °C. In-house STR DNA profiling analysis was used to authenticate the cell lines, and mycoplasma contamination was assessed. All clinical samples (49 ovarian cancer tissues and 12 normal ovarian tissues) were obtained from the Department of Obstetrics and Gynecology of The University of Hong Kong at Queen Mary Hospital. The specimens were snap-frozen immediately after collection and were stored at −80 °C until use.

### Plasmids and cell transfection

The miR-141-expressing construct (pmR-ZsGreen1-miR-141) (Forward -TTGAGCTGAAAGCTTTGCACAAGG, Reverse-AAGTGACTTGGATCCG, 583 bp) was generated by PCR amplification and ligated into the pmR-ZsGreen1 plasmid (Clontech, Mountain View, CA). The KLF12-expressing construct (pCMV6-KLF12) and Sp1-expressing construct (pCMV6-Sp1) were purchased from Origene (Origene Technologies, Inc., Rockville, M.D., USA). The Ambion® Anti-miR™ miRNA Inhibitor targeting miR-141 was purchased from ThermoFisher (ThermoFisher Scientific, Waltham, MA, USA). A TriFECTa® Kit with 3 siRNAs (KD1: NM_007249 duplex 1, KD2: NM_007249 duplex 2, KD3: NM_007249 duplex 3) and shRNA (NM_007249.4) targeting KLF12 were purchased from Integrated DNA Technologies (IDT, Commercial Park, Coralville, IA, USA) and GeneCopoeia (GeneCopoeia, Inc., USA). The universal negative control RNA duplex (NC1), scrambled siRNA control and shRNA scrambled control clone for psi-HIV-U6 were used as the negative controls. For luciferase reporter assays, pairs of oligonucleotides containing the 3'UTR binding site for miR-141 were purchased from IDT. Cell transfection was performed using Lipofectamine™ 2000 (Invitrogen) according to the manufacturer’s instructions. Stable clones overexpressing miR-141 were harvested after approximately 2 weeks of G418 selection. The transiently silenced KLF12 cells were collected at 48 h post-transfection, and lentivirus was used to infect cancer cells with KLF12 shRNA.

### RNA extraction and quantitative real-time PCR

TRIzol reagent (Invitrogen, Life Technologies) was used to extract total RNA from cell lines and primary cancer tissue samples. Twenty-five nanograms of total RNA was used as a template to generate first-strand cDNA with miRCURY LNA™ Universal RT microRNA PCR using a Universal cDNA Synthesis Kit (Exiqon, Vedbaek, Denmark). Quantitative real-time PCR (QPCR) was performed using a miRCURY LNA™ Universal RT miRNA PCR Kit and SYBR Green master mix (Exiqon, Denmark) in an ABI 7500 system (Applied Biosystems). The miR-141 probes (product no. 204504) and SNORD48, the reference gene (product no. 203903), were purchased from Exiqon. Each sample was assayed in triplicate, and the relative expression of miR-141 was normalized to SNORD48.

### Cell viability assays, cell migration and anoikis assays

Cell viability was measured by XTT assays, foci formation assays and soft agar assays. For the XTT assay, approximately 1000–2000 cells were seeded into each well of a 96-well plate. The full serum medium was changed to 1% serum medium after cell seeding for 24 h. Cell viability was measured using a Cell Proliferation Kit II (Roche Biosciences, Indianapolis, IN, USA) according to the manufacturer’s instructions. To perform the foci formation assay, cells were seeded in 6-well plates at a density of 800 cells/well. After seeding the cells for 24 h, the medium was exchanged with 1% serum medium. After approximately 1–2 weeks, the colonies that formed were fixed with 70% ethanol for 30 min before staining with 20% Giemsa stain (Sigma). The foci were counted. The soft agar assay was used to examine the anchorage-independent growth of ovarian cancer cells. Cancer cells with a density ranging from 500 to 1000 cells/well were embedded in a 0.6% DMEM-agarose layer on top of a 2% DMEM-agarose base. After 14 to 21 days, the viable colonies with >20 cells were counted. The cell migratory capacity of ovarian cancer cells was examined by the Transwell cell migration assay kits (Chemicon International, Inc., Temecula, CA) according to the manufacturer’s instructions. The migratory cells on transwell filters were visualized counted by bright-field microscopy (TE300, Nikon). For the anoikis assay, 1 × 10^6^ cells were cultured in the wells of 6-well low-attachment plates pre-coated with 1% agarose gel. After 48 h of suspension, cells were harvested for cell death analysis by TUNEL assays and flow cytometry. Cleavage of PARP and cleavage of caspase3 were evaluated by western blotting. The results were generated from more than three separate experiments and are presented as the mean ± SD.

### Computational miRNA target prediction and RNA gene expression profiling

Three online programs, TargetScan 6.0 (http://www.targetscan.org/), PicTar (http://pictar.mdc-berlin.de/) and DIANA LAB (DIANA-microT web server v5.0, http://diana.imis.athena-innovation.gr/DianaTools/index.php?r=MicroT_CDS/index), were used to identify potential targets of miR-141. For the gene expression profiling, total RNA was extracted from KLF12 knockdown OVCA433 cells and the vector control cells. OD_260/280_ ratios (1.7-2.1) and RNA integrity number (RIN > 0.8) of the RNA samples were analyzed by a spectrophotometer and bioanalyzer, respectively. After verifying the quality, the samples were subjected to Affymetrix GeneChip processing (Human Genome U133 Plus 2.0 Array) in the Centre for Genomic Science, The University of Hong Kong. The GeneChip experiment labels RNA targets and hybridizes targets to the arrays, followed by stringent washes, staining and scanning. The data were analyzed by GeneSpring 12.6 (Agilent Technologies, Santa Clara, USA).

### Sp1 binding site prediction, Chromatin immunoprecipitation (ChIP) and Luciferase reporter assays

Sp1 motif (MA0079.3) in JASPAR CORE Vertebrata database is represented in terms of the Position Weight Matrix (PWM), as annotated in the TRANSFAC® database. It was used to scan Sp1 binding sites (BS) in BIRC5 promoter, +/−1 k to TSS, chr17:76209278–76211277 (based on hg19 genome assembly), and ten putative BS were found. Five of them are covered by Sp1 ChIP-Seq peaks in 2 studies from ENCODE (Sp1 ChIP-Seq in A549 and HCT116), and two of the five are even covered by Sp1 ChIP-Seq peaks in 3 more studies from ENCODE (Sp1 ChIP-Seq in GM12878, H1-hESC and HepG2).

The Chromatin immunoprecipitation (ChIP) Assay Kit (Millipore, Billerica, MA, USA) was used to investigate the competition binding of Sp1 and KLF12 on human survivin promoter. The immunoprecipitated chromatin was examined by PCR and the ChIP primers targeted the survivin promoter is shown (Additional file 1: Figure S3).

Luciferase constructs were generated by ligating oligonucleotides containing wild-type (KLF12, sense: 5'-CTCCCCTTCACAGTGTTAACACAAAAGGGC ATCACCATCCCACGATGTCTGT-3'; antisense: 5'-CTAGACAGACATCGTG GGATGGTGATGCCCTTTTGTGTTAACACTGTGAAGGGGAGAGCT-'’; E2F3, sense: 5'-CATGGAACCAGAACATCTGTCATGCAGTGTTGTCCCTT CCTACCTTCTTCCT-3'; antisense: 5'-CTAGAGGAAGAAGGTAGGAAGGG ACAACACTGCATGACAGATGTTCTGGTTCCATGAGCT-3'; MAP2K4 sense: 5'-CGT AATACCTGATTGATCACACAGTGTTAGTGCTGGTCAGAG AGACCTCATT-3'; antisense: 5'-CTAGAATGAGGTCTCTCTGACCAGCACT AACACTGTGTGATCAATCAGGTATTACGAGCT-3') or mutant (KLF12, sense: 5'-CTCCCCTTCACAGAGAAAACACAAAAGGGCATCACCATCCCA CGATGTCTGT-3'; antisense: 5'-CTAGACAGACATCGTGGGATGGTGATGC CCTTTTGTGTTTTCTCTGTGAAGGGGAGAGCT-3') 3'UTR binding sites of miR-141 into the multi-cloning region of the pmirGLO Dual-Luciferase miRNA Target Expression Vector (Promega, Madison, WI, USA). HEK-293 cells were seeded into a 24-well plate and co-transfected with 100 ng pmirGLO wild-type or mutant plasmids together with the pmR-141 plasmid and the pmR-ZsGreen1 empty vector as a control (400 ng in total) using Lipofectamine™ 2000 (Invitrogen). Luciferase activity was detected with a Dual-luciferase Assay Kit (Promega) 48 h post-transfection using a F-4500 fluorescence spectrophotometer (Promega). For the dose-dependent model, 200, 400 and 600 ng of pmR-141 and constant amounts of the KLF12 plasmid (200 ng) were co-transfected into HEK-293 cells. *Renilla* luciferase activity was used as the reference to normalize transfection efficiency. All experiments were repeated three times.

### Western blotting and human apoptosis array

Cells were harvested and lysed using lysis buffer (Cell Signaling Technology) containing protease inhibitors (Sigma) and phenylmethylsulfonyl fluoride (PMSF) (Sigma Chemical Co., St Louis, MO, USA). Equal amounts of protein were separated by 10% SDS-PAGE and then transferred to Immobilon-P Transfer Membranes (Millipore Corporation, Bedford, MA, USA). The membranes were pre-blotted in 5% skim milk prior to incubation in 1% skim milk containing primary anti-Sp1 (1:500; Millipore Darmstadt, Germany), anti-KLF12 and anti-XIAP (1:1000; Santa Cruz Biotechnology, Inc., Santa Cruz, CA, USA), anti-cleaved-PARP, anti-cleaved-caspase3 (1:1000; Cell Signaling Technology, Inc., Danvers), anti-DDK (1:1000) (OriGene Technologies, Rockville, MD) and anti-β-actin (1:10000; Sigma-Aldrich, St. Louis, MO) antibodies overnight. The membranes were incubated with horseradish peroxidase-conjugated goat anti-rabbit or anti-mouse IgG (Amersham) and visualized using ECL^TM^ Western Blotting Detection Reagent (Amersham). Images were captured by Fuji Medical X-Ray Film (Fuji) and developed by the Fuji system. The Human Apoptosis Array Kit (R&D Systems, Inc., USA) was used based on the manufacturer’s instructions.

### Immunohistochemistry (IHC) and miRNA locked nucleic acid (LNA) in situ hybridization (ISH)

*In situ* hybridization (ISH) was performed to validate miR-141 expression in a commercial ovarian cancer tissue array (OVC1021) (5 normal/benign samples and 97 cases of ovarian cancer) (Pantomics Inc., CA, USA) using the miRCURY LNA™ microRNA ISH Optimization Kit 5 (FFPE) (Exiqon, Vedbaek, Denmark) as described in our previous study [22]. First, the tissue assay was deparaffinized and incubated for 40 min at 37 °C with 20 μg/ml proteinase K. Then, the array was dehydrated followed by hybridization with a miR-141 probe (5'-DIG/CCATCTTTACCAGACAGTGTTA/DIG-3', 1:500) overnight at 50 °C. Next, anti-DIG reagent (sheep anti-DIG-AP, 1:400) was added, and the slide was incubated for 60 min at room temperature. Then, AP substrate was freshly prepared and applied to the slide for a 2 h incubation at 30 °C in a humidifying chamber, avoiding the dark. Finally, a nuclear counterstain was applied, and the slides were mounted with mounting medium (Eukitt).

For the immunohistochemistry analysis, xylene and alcohol at different percentages were utilized for slide deparaffinization and rehydration. Slides were then immersed in sodium citrate buffer (pH6) and boiled for 20 min. Inhibition of the endogenous peroxidase was carried out by applying 0.3% hydrogen peroxidase (H_2_O_2_). The slides were incubated with an anti-KLF12 polyclonal antibody (1:600) at 4 °C overnight after blocking with 10% normal rabbit serum for 45 min. A standard streptavidin-biotin-peroxidase complex method was used for staining, followed by counterstaining with Mayer’s hematoxylin. The stained slides were reviewed by two independent investigators. The results were analyzed by light microscopy, and scores were given based on the intensity and the percentage of the stained tissues.

### In vivo studies

For the intraperitoneal model, stable SKOV3 miR-141-expressing clones, or A2780cp shSu knockdown clone and the scrambled controls (2 × 10^6^) were intraperitoneally injected into 5-week-old female BALB/cAnN nude mice (or *N* = 5 per group for SKOV3 miR-141 or A2780cp shSu, respectively). All mice were sacrificed after 2 to 4 weeks, and intraperitoneal tumor nodules were extracted and weighed to compare relative tumor burden.

### Statistical analysis

Statistical analysis was performed using SPSS 14.0 (SPSS). A receiver operating characteristic (ROC) curve was applied to determine the cut-off points for the ISH and IHC data. Student’s *t-*test (for parametric data) and the Mann–Whitney test (for non-parametric data) were used. The results are expressed as the mean ± SD from at least three independent experiments. The χ2 test or Fisher’s exact test was used to analyze the association of miR-141 and KLF12 expression and the clinicopathological parameters. The Pearson correlation was used to show the inverse relationship of miR-141 and KLF12. *P* < 0.05 was regarded as statistically significant.

## Results

### miR-141 is frequently upregulated in ovarian cancer

**Fig. 1:**
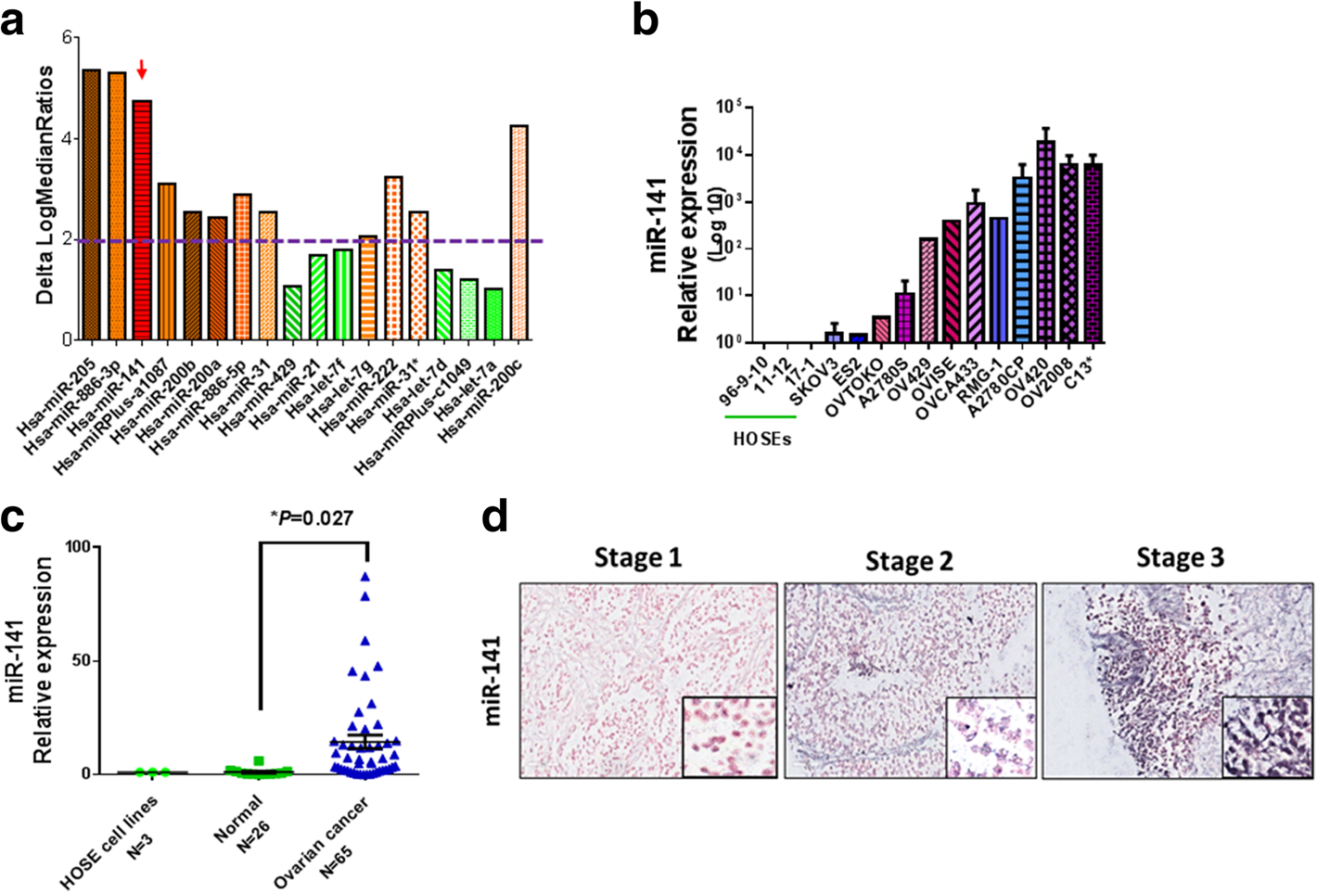
Mir-141 is frequently upregulated in advanced ovarian cancers. **a** A graph showing 18 miRNAs with the highest expression in microRNA profiling analysis which was performed using the miRCURY LNA™ miRNA array (Exiqon) on two types of ovarian cancer cell lines; anchorage-growth independent (A2780s, A2780cp, SKOV3, C13*), and anchorage-growth dependent (HOSEs). The dot line shows the miRNAs with more than 2 folds of Delta LogMedianRatios were selected for further functional analysis using transfection of their pre-miRNA expressing plasmids in ovarian cancer cell lines e.g. A2780cp and SKOV3 and performing of soft agar assay. **b** Real time qPCR analysis showed that miR-141 is upregulated in ovarian cancer cell lines (*N* = 12) as compared with the immortalized cell lines, HOSEs, (*N* = 3). Samples were normalized with *SNORD48*. **c** Graphical representation of qPCR analysis showing that miR-141 is elevated in ovarian cancer (*N* = 65) when compare to the control normal ovary tissues (*N* = 26) and immortalized cell lines (*N* = 3). **d** Representative pictures showing miR-141 ISH expression signal (dark blue staining) is gradually increased along the tumor stages of epithelial ovarian cancer. (Magnification × 10; scale bar, 100 μm)

The loss of anchorage dependence is the main consequence of tumor cell escape from detachment-induced cell death (anoikis). Using miRCURY™ LNA Array profiling (1264 miRNAs) analysis of two types of ovarian cancer cells (+/− in the ability to form colonies in soft agar assays), we found 18 upregulated miRNAs in the cell lines that could form colonies in soft agar (Fig. 1a). Among these 18 miRNAs, 12 potential candidates with higher than 2-fold changes in the median log ratios were selected for further functional screening. Soft agar assays were performed with ovarian cancer cells ectopically expressing the selected pre-miRNAs. The results showed that miR-141 significantly increased the anchorage-independent growth of ovarian cancer cells. However, other miR-200 family members, such as miR-200b/200c/429 and miR-200a, also showed high expression levels in ovarian cancer cells (Fig. 1a) but had no obvious anchorage-independent effects (data not shown), indicating that only miR-141 of the miR-200 family is involved with promoting anoikis resistance in ovarian cancer cells. The miRNA microarray findings were confirmed by comparing HOSE cell lines and ovarian cancer cell lines. Real-time quantitative RT-PCR (qPCR) was employed to assess a panel of ovarian cancer cell lines (SKOV3, ES2, OVTOKO, A2780S, OV429, OVISE, OVCA433, RMG-1, A2780cp, OV420) and three immortalized HOSE cell lines (HOSE17-1, HOSE 96-9-10 and HOSE 11–12). Our data showed that miR-141 was frequently upregulated in the ovarian cancer cell lines (*N* = 12) and ovarian cancer clinical samples (*N* = 49) compared to either normal ovarian tissues (*N* = 12) or HOSE cells (*N* = 3) (Fig. 1b & c). Furthermore, consistent with the findings in the cell lines, the mean expression level of miR-141 in clinical ovarian cancer samples (*N* = 49) was found to be ~10-fold higher than that of normal ovary tissues (*N* = 12) as well as the HOSE cell lines (Fig. 1c). *In situ* hybridization was then performed on a commercial human ovarian cancer tissue array (OVC1021, Pantomics), which further confirmed that the upregulation of miR-141 in advanced ovarian cancer is significantly correlated with cancer metastasis (Fig. 1d, Table 1). These findings suggest that upregulation of miR-141 is common in ovarian cancers, especially late-stage ovarian cancer.

**Table 1:** Clinicopathological correlation of the expression of miR-141 in an ovarian cancer tissue array (OVC1021). The 5-fold cut-off was determined by ROC analysis

Parameters	n(=97)	*miR-141* expression		
		≤5-fold	>5-fold	*p*
Grade				
Low (1 + 2)	50	24 (48%)	26 (52%)	
High (3)	46	24 (52%)	22 (48%)	0.838
Stage				
Early (1)	48	31 (65%)	17 (35%)	
Late (2 + 3)	49	18 (37%)	31 (63%)	0.008*
Metastasis				
Yes	24	5 (21%)	19 (79%)	
No	73	44 (62%)	29 (38%)	0.001*

*P < 0.05

### Increased miR-141 augments anchorage-independent growth and survival of ovarian cancer cells in vitro and tumor growth in vivo

**Fig. 2:**
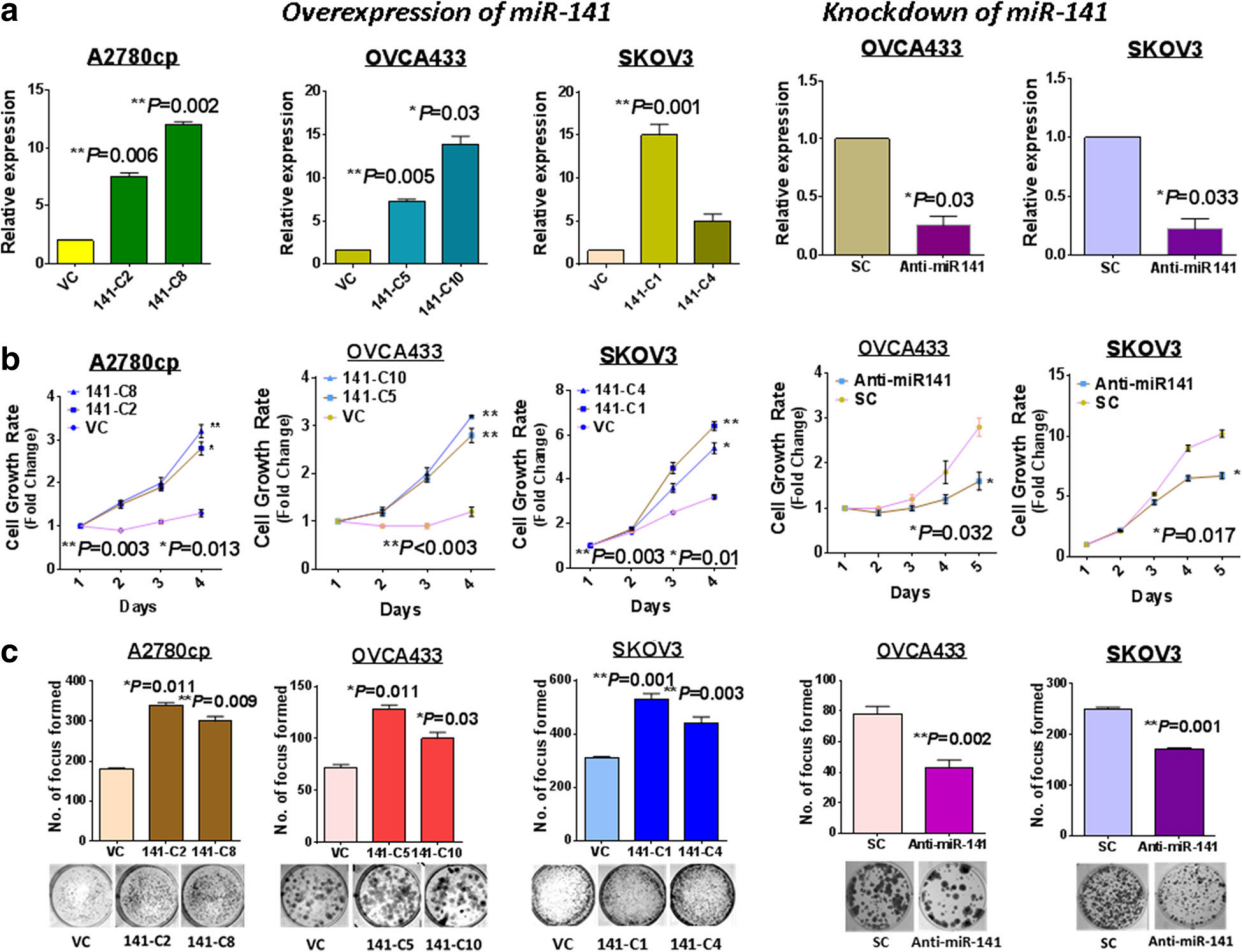
miR-141 enhances ovarian cancer cell survival. QPCR analyses showed the expressions of miR-141 in, (**a**) (*left*) miR-141-overexpressing clones of A2780cp, OVCA433 and SKOV3; (*right*) and anti-miR141 treated cell lines; OVCA433 and SKOV3. VC: empty control and SC: scrambled control. **b** (*left*) XTT cell proliferation demonstrated that overexpression of miR-141 could remarkably enhanced the cell proliferation in A2780cp, OVCA433 and SKOV3 cell lines; (*right*) while inhibition of miR-141 significantly attenuated the cell proliferation in OVCA433 and SKOV3 cells with treatment of anti-miR141. The foci formation assay demonstrated that, (**c**) (*left*) overexpression of miR-141 could increase the cell viability in low-serum medium (0.1% FBS) compared with the vector control, (*right*) while depletion of miR-141 led to a reduction of cell viability

Upregulated miR-141 was strongly associated with advanced ovarian cancers, which is consistent with a previous study [23]. To confirm the oncogenic functions of miR-141, a miR-141 precursor-expressing plasmid (pmR-ZsGreen1 vector, Clontech) was stably transfected into three advanced ovarian cancer cell lines: A2780CP (p53 mutated) (141-C2 and 141-C8), OVCA433 (p53 wildtype) (141-C5 and 141-C10) and SKOV3 (p53 deleted) (141-C1 and 141-C4) (Fig. 2a, *left panel*). An ability to survive in the stressful microenvironment is one of the pre-requisites for the development of anoikis resistance in cancer cells. Therefore, we examined the cell viability of stable miR-141-expressing clones under low serum conditions (1% FBS). Using XTT cell proliferation assays, we showed that all miR-141-expressing clones had significantly higher proliferation rates (3- to 4-fold) than the scrambled controls (Fig. 2b, *left panel*). Furthermore, foci formation assays also confirmed that stable miR-141-expressing clones of the three cell lines exhibited increased cell viability (Fig. 2c, *left panel*) under low serum (1%) culture conditions. The ability to survive in unfavorable conditions provides advantages to cancer cells in acquiring anoikis resistance. To evaluate cancer cell resistance to anoikis, soft agar assays were used. Stable miR-141-expressing clones of A2780cp (141-C2 and 141-C8) and SKOV3 (141-C2 and 141-C3) were tested in this assay. Our results revealed that the clones A2780cp (141-C2 and 141-C8) exhibited a substantial increase in size (~10-fold) and number (1.5- to 2-fold), and the SKOV3 stable clones (141-C2 and 141-C3) also exhibited an increase in size (~2- to 4-fold) and number (1.5- to 2-fold) in soft agar compared with the vector controls (Additional file 2: Figure S1).

Then, miR-141 was inhibited by the anti-miR™ miRNA Inhibitor (Ambion) in the miR-141-enriched OVCA433 and SKOV3 cells (Fig. 1b). The depletion of miR-141 in OVCA433 and SKOV3 (Fig. 2a, *right panel*) led to a significant reduction in cell viability by 38 to 45% (Fig. 2b, *right panel*) and the number of foci by 45 to 65% (Fig. 2c, *right panel*) in the ovarian cancer cells cultured in low serum medium. Taken together, these findings suggested that miR-141 plays a crucial role in mediating cell survival under stress condition in ovarian cancer cells.

### KLF12 is a direct target of miR-141 in ovarian cancer

**Fig. 3:**
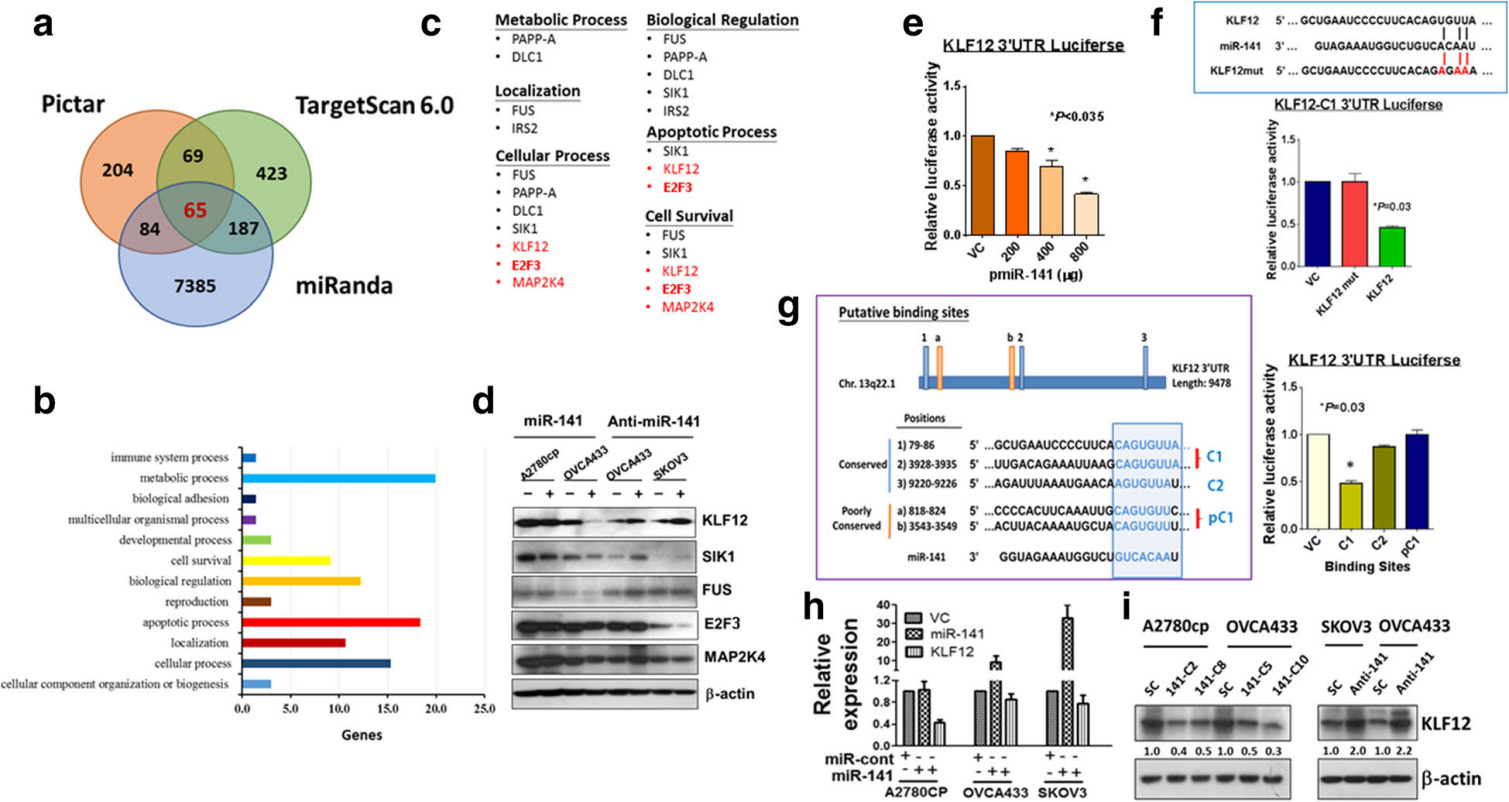
KLF12 is a direct target of miR-141. **a** The downstream targets of miR-141 were predicted by an *in silico study* using three bioinformatics algorithms (PicTar, Miranda, and TargetScanHuman 6.0). **b** Ontology analysis using miRNA_Targets database 22 identified those predicted targets are involved in metabolic process, biological regulation, localization and cellular process etc. **c** The putative miR-141 targets which may be associated with anoikis resistance of ovarian cancer cells are categorized into six functional aspects. The red highlighted genes are the novel targets, while the black color genes have been reported as the miR-141 direct targets. **d** Western blot analysis showed that enforced expression of miR-141 (+) could reduce, while depletion of endogenous miR-141 by anti-miR-141 (+) could enhance the expressions of KLF12 and SIK1 only as compared with their controls (−) in ovarian cancer cells. But the expressions of other putative downstream targets; FUS, E2F3 and MAP2K4 had little or no change when the level of miR-141 was altered. **e** Luciferase reporter assay was performed in HEK293 cells and showed that the 3’ UTR of KLF12 luciferase activity was significantly inhibited by miR-141 expression dependently. **f** A schematic diagram shows the putative binding sites for miR-141 on the whole 3'UTR region of KLF12. Transient transfection of miR-141 mitigated the expression of KLF12 3’UTR-luciferase reporter activity. The KLF12 3’UTR with C1 binding site showed the highest suppression by miR-141. **g** (*Left panel*) The schematic diagram shows the most conserved and common pairing of wild-type and its mutant target regions of KLF12 and miR-141. (*Right panel*) The bar chart shows that miR-141 remarkably reduced the wild-type, whereas the KLF12_3’UTR mutant showed no change in luciferase reporter activity. **h** QPCR showed that miR-141 could reduce the expression of KLF12 mRNA in ovarian cancer cell lines. **i** Stable expression of miR-141 significantly reduced the expression of KLF12 in A2780cp and OVCA433 cells, while depletion of miR-141 using anti-miR-141 restored the expression of KLF12 in SKOV3 cells

One miRNA can modulate a wide range of downstream targets; thus, we examined the potential downstream targets of miR-141 that may mediate these tumorigenic behaviors in ovarian cancer cells. Through an *in silico* study using three bioinformatics algorithms (PicTar, Miranda, and TargetScanHuman 6.0), we successfully sorted out 65 potential genes from the above 3 programs using gene ontology terms according to the Database for Annotation, Visualization and Integrated Discovery (DAVID) [24] (Fig. 3a). Ontology analysis using miRNA_Targets database [25] identified that those potential targets are involved in metabolic process, biological regulation, localization and cellular process (Fig. 3b). Notably, some potential genes have been previously reported that they are the direct targets of miR-141 and are associated with cell proliferation and metastasis of human cancers. For examples, PAPP-A, a factor of metabolic process, is frequently found in invasive breast cancer [26, 27]; FUS, a binding protein, is associated with neuroblastoma’s proliferation, migration and survival [28]; DLC1 is a well-known tumor suppressor for a variety of human cancers [29]; as well as IRS2 is a suppressor of cell proliferation and invasion of thyroid cancer [30]. Interestingly, there is a potential target, SIK1, has previously reported to be associated with anoikis resistance and metastasis via interaction with LKB1 and in p53-dependent manner [31]. Likewise, our findings demonstrated that increased miR-141 significantly enhances the anoikis resistance of ovarian cancer cells. Among these cell lines, some cell lines, such as A2780cp and SKOV3, harboring mutated or deleted p53, suggesting that the increased anoikis resistance of ovarian cancer cells does not solely depend on SIK1/LKB1/p53 axis. To explore other putative targets associated with anoikis resistance in ovarian cancer cells, we next analyzed other potential targets which are involved in cell survival and have high score in algorithms prediction. In line with two known targets of miR-141; SIK1 and FUS, we also evaluated the expression of another three putative targets; E2F3, MAP2K4 and KLF12 (Fig. 3c). Our western blot analysis showed that overexpression of miR-141 and transfection of anti-miR141 could remarkably reduce and increase the expressions of SIK1 and KLF12, respectively (Fig. 3d). However, FUS, MAP2K4 and E2F3 just showed minor or even no change in the expressions (Fig. 3d). To further confirm these three putative targets was controlled by miR-141, the 3'UTR regions flanking the miR-141 binding sites of each target were subcloned into a luciferase reporter vector, pmirGLO, generating pmirGLO-MAP2K4, pmirGLO-E2F3 and pmirGLO-KLF12. Luciferase reporter assays of cells co-transfected with miR-141 and the three target plasmids revealed that the E2F3 and MAP2K4 vectors only showed a 10% reduction in luciferase reporter activities (*P* < 0.005), while KLF12 showed a 35% reduction (*P* < 0.001) compared with the vector control (Additional file 3: Figure S2). This finding is in line with the above western blot analysis (Fig. 3d), indicating E2F3 and MAP2K4 may not the direct targets of miR-141. On the other hand, further analysis indicated that miR-141 could significantly reduce the pmirGLO-KLF12 luciferase activity in a dose-dependent manner (*P* < 0.035) (Fig. 3e). There are 3 conserved (2 types of miR-141 target sequence) and 2 poorly conserved (one type of miR-141 target sequence) miR-141 binding sites in the KLF12 3'UTR (Fig. 3f). Using luciferase reporter assays, we found that miR-141 could significantly attenuate the luciferase activity from the reporter fused to the C1 miR-141 target sequence (GTCACAAT) of the KLF12 3'UTR by 51% and the C2 miR-141 target sequence (TCACAAT) by 14%, while there was no effect on the pC1 poorly conserved miR-141 target sequence (CAGTGTT) (Fig. 3g). Additionally, a point mutation (AACCAAT) in the miR-141 seed region in the 3'UTR (pmiRGLO-KLF12-MUT) abolished the suppressive effect on luciferase activity (Fig. 3g). To determine whether there is an inverse correlation between miR-141 and KLF12 expression, the ovarian cancer cell lines OVCA433, A2780CP and SKOV3 were transiently transfected with miR-141. The qPCR analysis confirmed the ectopic expression of miR-141 in the cell lines (1- to 30-fold), while a simultaneous reduction in KLF12 expression of 20 to 60% was observed (Fig. 3h). Moreover, western blotting also confirmed that the expression levels of KLF12 and miR-141 were inversely correlated in miR-141 overexpressed OVCA433 and A2780CP cells, and miR-141-depleted SKOV3 and OVCA433 cells using anti-miR™ miRNA Inhibitor (Fig. 3i). These results suggest that miR-141 regulates a network of genes or signaling inside the mammalian cells, while KLF12 is a direct downstream target of miR-141, and miR-141 could inhibit the expression of KLF12 mRNA and protein levels.

### KLF12 exerts a tumor-suppressive effect on ovarian cancer cells

**Fig. 4:**
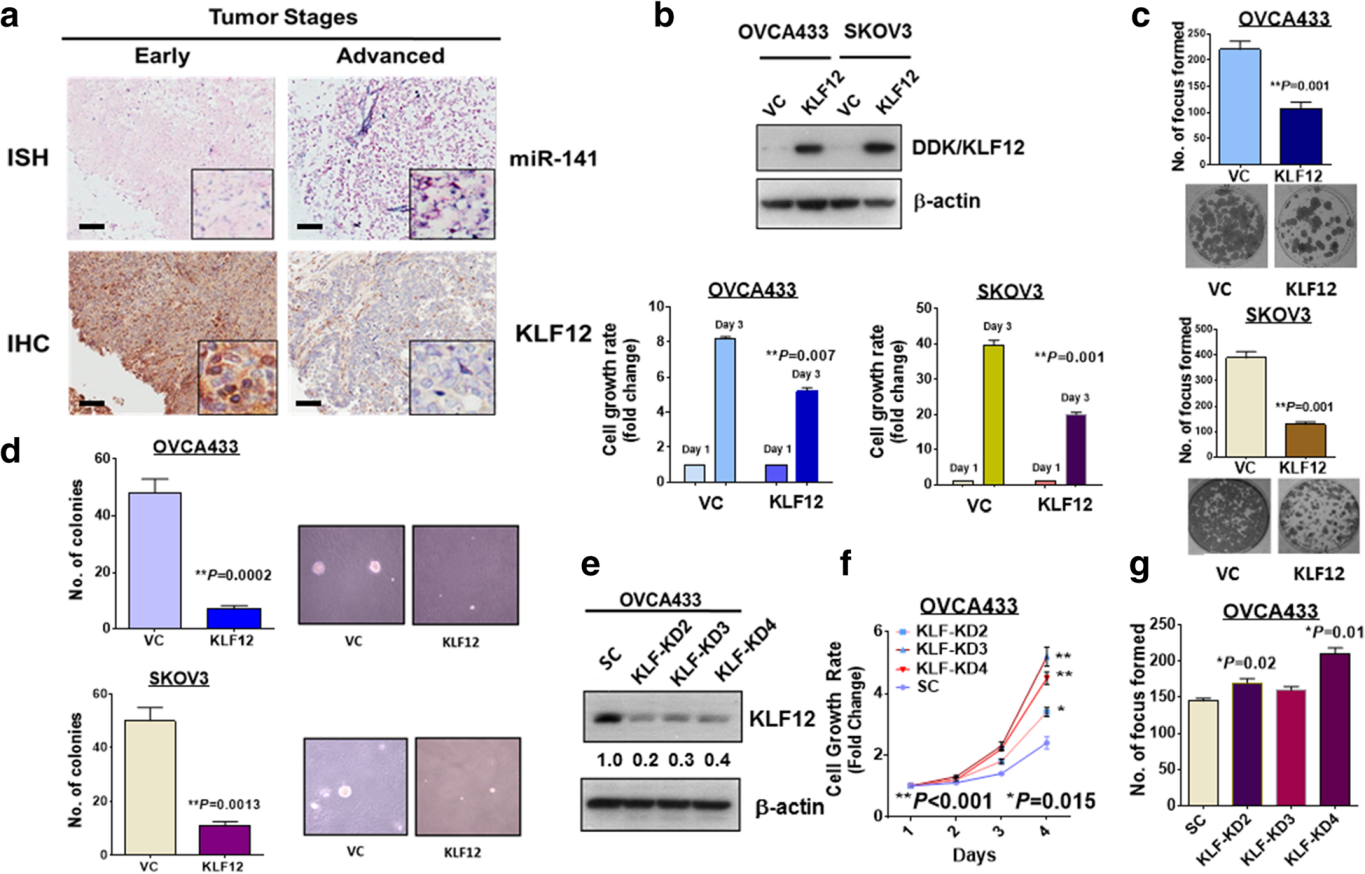
KLF12 expression inversely correlates with miR-141 and exerts inhibitory effects on ovarian cancer tumorigenicity. **a** Representative pictures showing miR-141 expressions examined by ISH (dark blue staining) is inversely correlated with KLF12 expression detected by IHC (brown staining) in early and advanced ovarian cancers. (Magnification × 10; scale bar, 100 μm). **b** Overexpression of KLF12 in two ovarian cancer cells; OVCA433 and SKOV3 (*Upper*), significantly suppresses their cell growth rates (Day 3 vs Day 1) examined by XTT cell proliferation assay when compared the cell growth rate of their empty vector controls, VC. **c** Focus formation assay revealed that overexpression of KLF12 significantly inhibit the number of foci in OVCA433 and SKOV3 cultured in low serum medium for 14 days. **d** The soft agar assay showed that overexpression of KLF12 remarkably reduce the number and size of colonies formed in soft agar in OVCA433 and SKOV3 cells as compared with their empty vector controls. The above assays represent the error bars with mean ± SD of at least 3 independent experiments. **e** Western blot analysis revealed that three KLF12 stable clones were obtained from transfection of shRNAi of KLF12 in OVCA433. SC represents scrambled control. These results were obtained from at least three experiments. **f** XTT cell proliferation assay showed that there was a significant increase of cell growth rate in all three KLF12 knockdown clones as compared with the scramble control (SC). **g** The focus formation assay showed that there were two KLF12 knockdown clones (KD2 and KD4) had a significant increase in the number of foci

As there is an inverse relationship between KLF12 and miR-141, KLF12 may play an opposing role in ovarian cancer cell tumorigenicity and may have relatively low expression. Immunohistochemistry (IHC) was performed on the same human ovarian cancer tissue array used for the evaluation of miR-141 expression by ISH (OVC1021, Pantomics). Together with the ISH findings, the IHC results demonstrated that high expression of miR-141 was significantly associated with low expression of KLF12 (*N* = 97, *P* < 0.001) (Fig. 4a) (Tables 1 and 2). Notably, clinicopathological analysis showed that both high expression of miR-141 (>5-fold) and low expression of KLF12 (≦5-fold) were significantly correlated with late-stage cancers (*P* = 0.008 for high expression of miR-141 and *P* = 0.000 for low expression of KLF12) and metastasis (*P* = 0.001 for high expression of miR-141 and *P* = 0.01 for low expression of KLF12) (Tables 1 and 2). Further study by analyzing Pan-Cancer miRNA-mRNA interaction maps from the Cancer Genome Atlas (TCGA) matched miRNA-Seq and RNA-Seq data using StarBase V2.0 [32, 33] (http://starbase.sysu.edu.cn/index.php), we found that the inverse correlation between miR-141 and KLF12 is not only observed in ovarian cancers but also other cancer types (11/14) (Additional file 1: Figure S3). This suggests that this inverse relationship between miR-141 and KLF12 is generally found in human cancers.

**Table 2:** Clinicopathological and miR-141 correlation of the expression of an ovarian cancer tissue array (OVC1021). The 5-fold cut-off was determined by ROC analysis

Parameters	n(=97)	KLF12 expression		
		≤5-fold	>5-fold	*p*
Grade				
Low (1 + 2)	50	25 (50%)	25 (50%)	
High (3)	46	24 (52%)	22 (48%)	0.841
Stage				
Early (1)	48	14 (29%)	34 (71%)	
Late (2 + 3)	49	36 (73%)	13 (27%)	0.000*
Metastasis				
Yes	24	18 (75%)	6 (25%)	
No	73	32 (44%)	41 (56%)	0.010*
miR-141				
≤ 5 folds	50	14 (28%)	36 (72%)	
> 5 folds	48	37 (77%)	11 (23%)	0.000*

*P < 0.05

On the other hand, overexpression of KLF12 showed a 1- to 2-fold reduction in cell growth in the OVCA433 and SKOV3 cells cultured in low serum media (Fig. 4b), a 2-fold reduction in foci formation in OVCA433 cells (Fig. 3c), and a 5- to 7-fold reduction in colony formation in soft agar assays for OVCA433 and SKOV3 cells (Fig. 4d). In contrast, stable knockdown of KLF12 in two highly expressing KLF12 cell lines, A2780cp and OVCA433 (Fig. 4e), showed a 0.2- to 2.5-fold increase in cell growth (Fig. 4f) and a 10% to 30% increase in foci formation (Fig. 4g). These findings demonstrate that KLF12 acts a downstream target of miR-141 and a tumor suppressor in ovarian cancers.

### miR-141 confers anoikis resistance by suppressing KLF12 in ovarian cancer cells

**Fig. 5:**
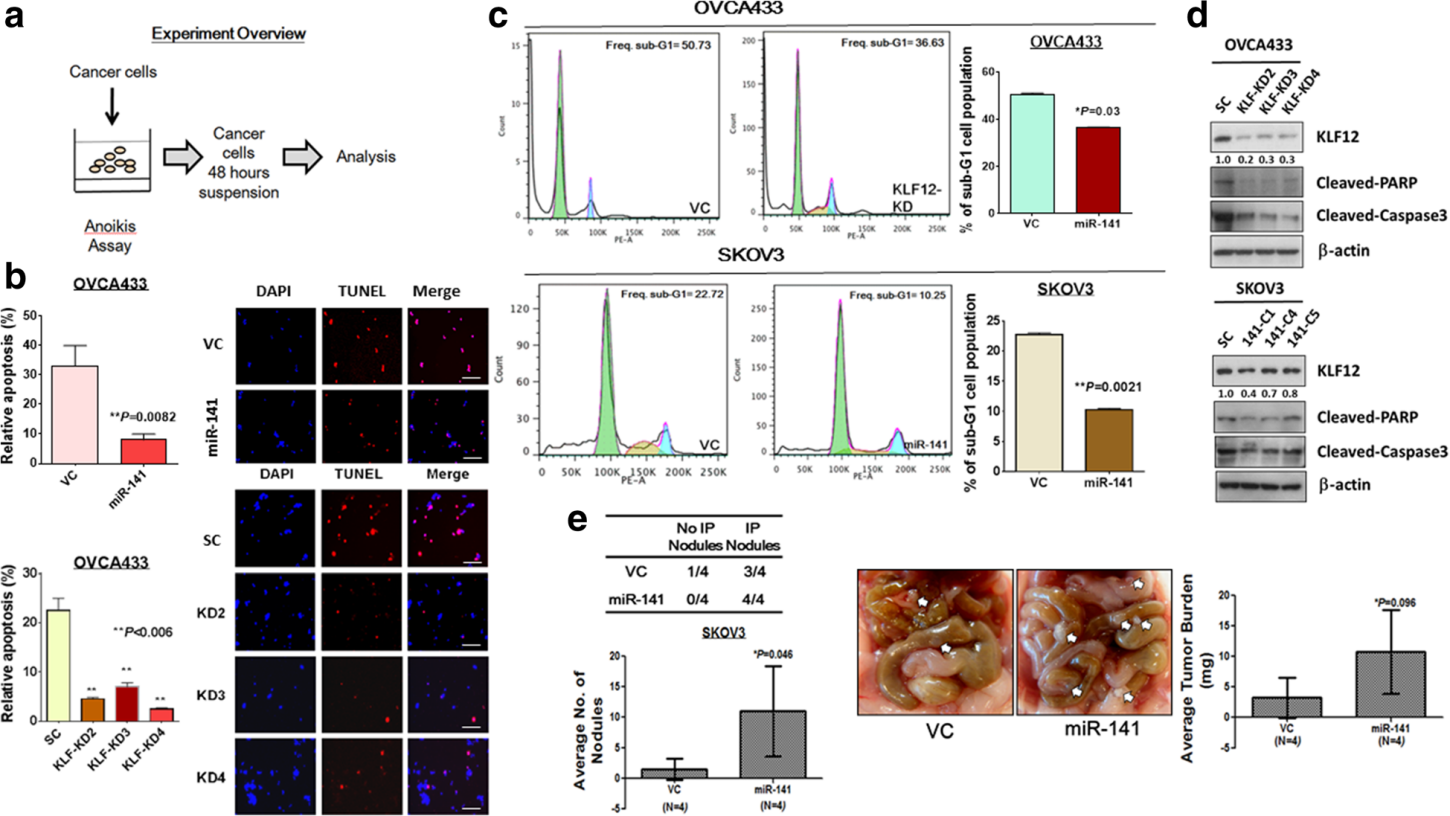
miR-141 promotes ovarian cancer cell growth and tumor seeding by enhancing anoikis resistance of ovarian cancer cells. **a** Experimental overview of anoikis assay. **b** The relative apoptosis rates of miR-141 stable clones and KLF12 knockdown cells were measured using TUNEL assay. Green signals represent DAPI positive cells and red signals represent TUNEL positive cells. Both overexpression of miR-141 or knockdown of KLF12 resulted in lower apoptotic rates of ovarian cancer cells after treated by anoikis assay. **c** Flow cytometry cell cycle analysis revealed that percentages of sub-G1 phase in either KLF12 knockdown OVCA433 cells or miR-141 overexpressed SKOV3 cells were reduced as compared with their negative controls. **d** Western blotting showed that either knockdown of KLF12 or overexpression of miR-141 could suppress ovarian cancer cell apoptosis through reduction of cleaved-PARP and cleaved-caspase 3 expression in anoikis treated ovarian cancer cells (*Upper*: KLF12 knockdown clones in OVCA433, *Lower*; miR-141 stable expressing clones in SKOV3). Bar, 50 μM. **e** Effects of miR-141 on ovarian cancer growth and tumor seeding were revealed using *in vivo* model by i.p. injecting miR-141 and the scrambled control clones into 5-weeks old female nude mice. Average number of tumor nodules (*Left*) and the tumor burden (*Right*) formed by miR-141 clones were much higher than the scrambled control and they were observed across the peritoneal cavity as photographed on Day 28 (4 weeks). The white arrows indicate the location of tumor nodules

Given that miR-141 is involved in promoting resistance to the stress microenvironment in ovarian cancer cells, we next investigated whether miR-141 increases anoikis resistance in ovarian cancer cells. Anoikis is a type of apoptosis induced by inappropriate cell-matrix interactions [34], and the anoikis assay is a method that mimics the microenvironment of the cells when the extracellular matrix attachment is absent [35] (Fig. 5a). Upon pre-treatment with the anoikis method, OVCA433 cells overexpressing miR-141 and with knockdown of KLF12 showed fewer apoptotic cells (red signals) (overexpressing miR-141, 3-fold; knockdown of KLF12, 3- to 8-fold) compared with their scrambled controls using TUNEL assays, indicating that there is a substantial reduction in apoptosis in OVCA433 cells (Fig. 5b). Flow cytometry analysis of KLF12 knockdown clones or miR-141-overexpressing clones further showed a reduction in the sub-G1 phase (~28% reduction for KLF12 knockdown clones of OVCA433 and ~56% for miR-141-overexpressing clones of SKOV3) (Fig. 5c), indicating less apoptosis in KLF12 knockdown OVCA433 cells or miR-141-overexpressing SKOV3 cells. Furthermore, western blotting revealed that expressions of apoptosis-related factors, cleaved-PARP and cleaved-caspase3, were reduced drastically (Fig. 5d). Collectively, the above results indicate that the overexpression of miR-141 not only increases cell viability in stress microenvironments but also confers anoikis resistance to the ovarian cancer cells by inhibiting KLF12.

To examine the functional role of miR-141 in metastatic peritoneal ovarian cancer growth and progression, miR-141 was stably expressed in SKOV3 clones (*N* = 4), and these cells and the vector controls (*N* = 4) were intraperitoneally (i.p.) injected into nude mice. After 4 weeks, tumor seeding and invasion were examined in a post-mortem examination. Although injection of both the vector control and the miR-141 clones resulted in tumor nodule formation, tumor seeding was observed in only three out of four mice in the vector control group while all four mice in the miR-141 group showed a greater extent of tumor burden. Moreover, compared with the vector control, cells with stably expressing miR-141 had a greater number of macroscopic tumor nodules studding throughout the omentum and the peritoneal cavity. Each mouse injected with the miR-141 clones formed an average of more than 10 intraperitoneal tumor nodules, while there was an average of approximately 2 nodules in the vector control group (Fig. 5e). Taken together, these results further suggest that in addition to promoting anoikis resistance, miR-141 also promotes metastatic ovarian cancer cell growth and tumor seeding in the peritoneal cavity, which is important in the metastatic process.

### miR-141 serves as a novel modulator of the intrinsic apoptotic pathway

**Fig. 6:**
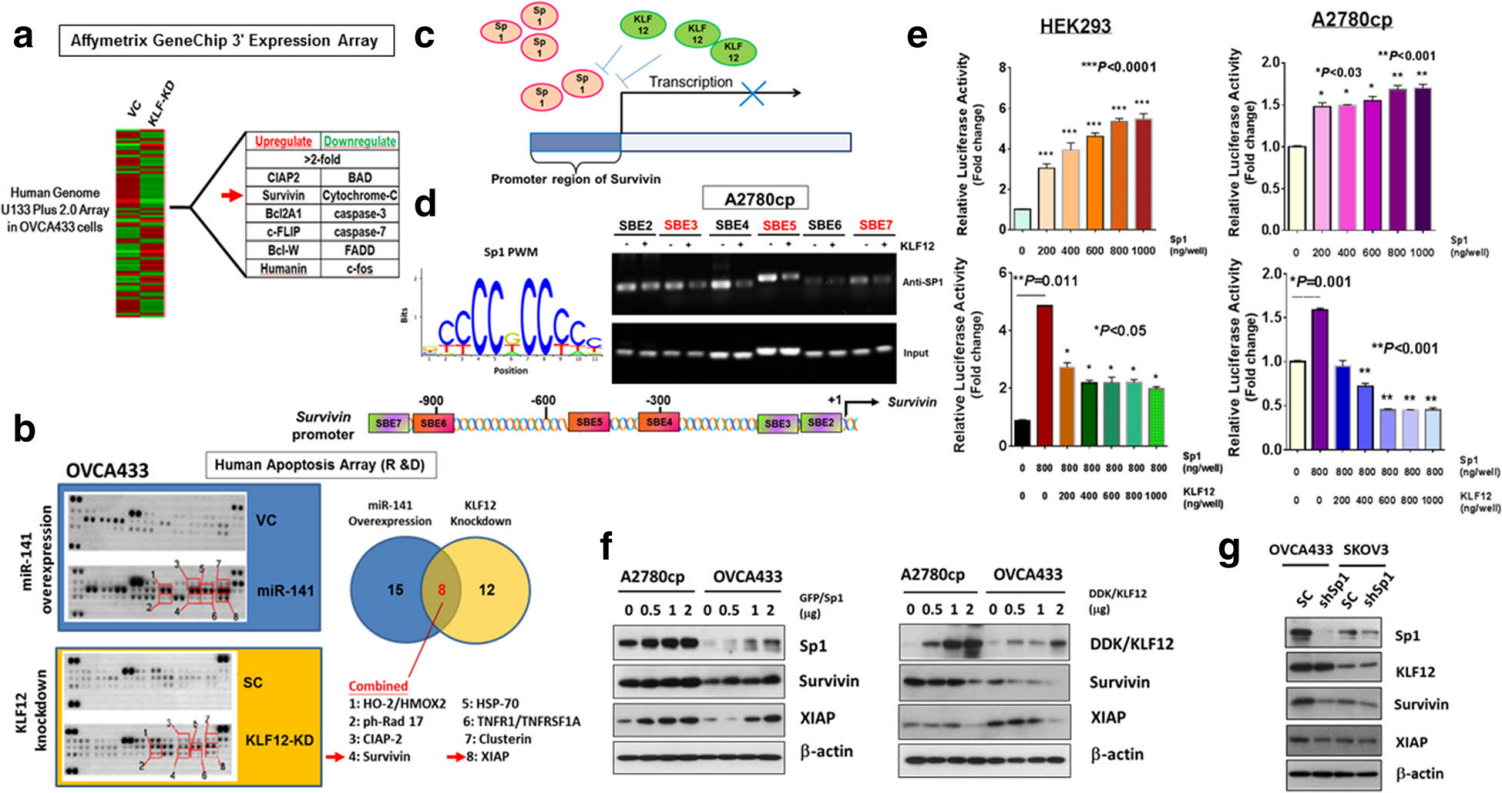
Increased anoikis resistance in ovarian cancer cells is involved intrinsic apoptotic pathway. **a** The Affymetrix GeneChip 3' Expression Array revealed a panel of apoptosis-related genes (Fold change >2) induced in KLF12 knockdown OVCA433 cells which showed increased anoikis resistance. **b** Proteome Profiler (Human Apoptosis Array Kit) showed that 8 out of 35 Intrinsic Apoptosis-related factors were commonly upregulated in miR-141 enforced expression or KLF12 knockdown OVCA433 cells. **c** A schematic diagram illustrates that there are several Sp1 binding sites located along the promoter of survivin, confirming Sp1 is able to transcriptionally upregulate survivin. KLF12 is able to interfere the interaction of Sp1 with the promoter of survivin and the transcriptional activity. **d** ChIP assay showed the competitive occupancy of Sp1 by KLF12 on the promoter of *survivin*. (*Upper*) Position weight matrix (PWM) for SP1-binding sequence motif derived from Jaspar database. ChIP assays showed the occupancy of Sp1 on 3 SBEs (*Red color*) of the *Survivin* promoter in A2780cp cells were inhibited by KLF12. (*Lower*) Schematic diagram shows the positions of seven higher binding affinity SBEs on the *survivin* promoter. **e** Luciferase reporter assay using Survivin promoter luciferase reporter construct (luc-Survivin) demonstrated that Sp1 could significantly elevate the luciferase reporter signals from ~3-5.5 folds in HEK293 and ~48 to 70% in A2780cp by Sp1 dose dependently (200 to 1000 ng/well) (*Upper*). Using luc-survivin and pCMV6-AC-GFP-Sp1 (600 ng/well) and co-transfected with varied amount of pCMV6-KLF12 (200–800 ng/well), results showed that the Sp1-upregulated survivin signals were remarkably reduced from 5 to 2 folds in HEK293, and from 158% to 45% in A2780cp by KLF12 in a dose-dependent manner (200 – 1000 ng/well). **f** Western blot analysis showed that transient transfection of Sp1 could upregulate, while KLF12 could inhibit, survivin and XIAP expressions in A2780cp and OVCA433 cells. **g** Western blot analysis confirmed that knockdown of Sp1 had no effect on KLF12 expression but significantly reduced the expression of survivin and XIAP in two Sp1-enriched ovarian cancer cell lines, OVCA433 and SKOV3

To decipher the underlying molecular mechanism associated with miR-141/KLF12-mediated anoikis resistance, we examined their downstream targets. KLF12 is a transcription factor that belongs to the Sp1/KLF family [36]. Affymetrix GeneChip 3' expression arrays were used with mRNAs isolated from OVCA433 KLF12 stable knockdown clones (KLF-KD4) to identify the downstream targets regulated by KLF12 (Fig. 6a). Using a fold change >2 as a threshold, we identified a panel of differentially expressed genes, particularly those related to the apoptosis. Intriguingly, most highly altered genes revealed by array profiling analysis were associated with anti-apoptotic effects, e.g., CIAP2, Bcl2A1 and survivin (Fig. 6a), supporting the hypothesis that KLF12 is crucial in combating anoikis during cell detachment. Furthermore, using a Human Apoptosis Array Kit consisting of 35 apoptosis-related proteins (R&D) with either miR-141-overexpressing or KLF12 knockdown ovarian cancer cells, 8 out of 35 proteins (HO-2/HMOX2, ph-Rad 17, CIAP-2, survivin, HSP-70, TNFR1/TNFRSF1A, clusterin and XIAP) were upregulated (Fig. 6b). Upregulation of survivin was found in both GeneChip and the Human Apoptosis Arrays (Fig. 6b). Survivin, a structurally unique IAP protein, is a key regulator of the intrinsic apoptotic pathway. Previous findings reported that survivin forms a complex with XIAP and inhibits the activation of caspase-3 and caspase-9, which in turn blocks the intrinsic apoptotic pathway [37] and prevents cell apoptosis due to anoikis stress. X-linked inhibitor of apoptosis protein (XIAP), also known as inhibitor of apoptosis protein 3 (IAP3), is also upregulated through association with the increased survivin [38]. Hence, we speculated that the miR-141-induced anoikis resistance is mediated through suppression of KLF12, which, in turn, upregulates survivin and XIAP expression to inhibit ovarian cancer cell apoptosis. Previous studies have suggested that the KLF family shares homology with the transcriptional factor Sp1, and therefore, they are grouped together in the Sp1/KLF family [39]. Thus, we hypothesized that KLF12 and Sp1, which share homology, may compete for binding sites on the survivin promoter, hampering the transcriptional activation of survivin (Fig. 6c). To test this hypothesis, we first used Sp1 motif from Jaspar database to search the Sp1-binding elements (SEBs) on the survivin promoter (Fig. 6d). Taking the promoter region (+/−1 k to TSS) of survivin: chr17:76209278–76211277, we found that ten putative SEBs (Additional file 4: Figure S4), showing higher potential of Sp1 binding according to recent findings [40]. By their affinity score and distribution on *survivin* promoter, 7 out of 10 binding sites were examined for the competition of SEBs occupancy between Sp1 and KLF12 by ChIP assay (Additional file 4: Figure S4). Results revealed that the occupancy of Sp1 on 3 out of 7 SBEs of the *survivin* promoter were obviously inhibited by KLF12 in A2780cp ovarian cancer cells (Fig. 6d), supporting our notion of that KLF12 compete for the binding sites of Sp1 on the *survivin* promoter and in turns hampers the transcriptional activity of *survivin*.

To further prove that, we next examined whether the transcriptional activity of survivin is upregulated by Sp1 using luciferase reporter assays. pCMV6-Sp1 and a survivin promoter luciferase plasmid (luc-survivin) (CH3 Biosystems) were co-transfected into HEK293 cells and A2780cp cells. The results showed that the luciferase activity of the survivin promoter was significantly increased by ~3- to 5.5-fold in HEK293 cells and by ~48 to 70% in A2780cp cells by Sp1 in a dose-dependent manner (Fig. 6e). In contrast, when luc-survivin and pCMV6-Sp1 (600 ng) were co-transfected with varied amounts of pCMV6-KLF12 (200–800 ng) in HEK293 and A2780cp cells, the luciferase reporter results showed that the Sp1-upregulated survivin signals were strongly reduced by KLF12 from 5-fold to 2-fold in HEK293 cells and from 158% to 45% in A2780cp cells in a dose-dependent manner (Fig. 6e). These results suggest that KLF12 has an opposing function to Sp1 and augments ovarian cancer cell survival by modulating the expression of survivin. To further verify these results, we transiently transfected A2780cp and OVCA433 cells with either Sp1 or KLF12. Western blot analyses indicated that Sp1 could significantly elevate the expression levels of survivin as well as XIAP, while KLF12 had the opposite effects compared to Sp1 and suppressed survivin and XIAP expressions in both ovarian cancer cell lines (Fig. 6f). In contrast, stable knockdown of Sp1 in OVCA433 and SKOV3 reduced the expression of survivin and XIAP, while the expression of KLF12 was not changed (Fig. 6g). These data suggest that the overexpression of miR-141 or the loss of KLF12 enhances anoikis resistance in ovarian cancer cells by modulating the Sp1/survivin/XIAP intrinsic apoptotic pathway.

### Survivin is required for enhancing anoikis resistance in ovarian cancer cells

**Fig. 7:**
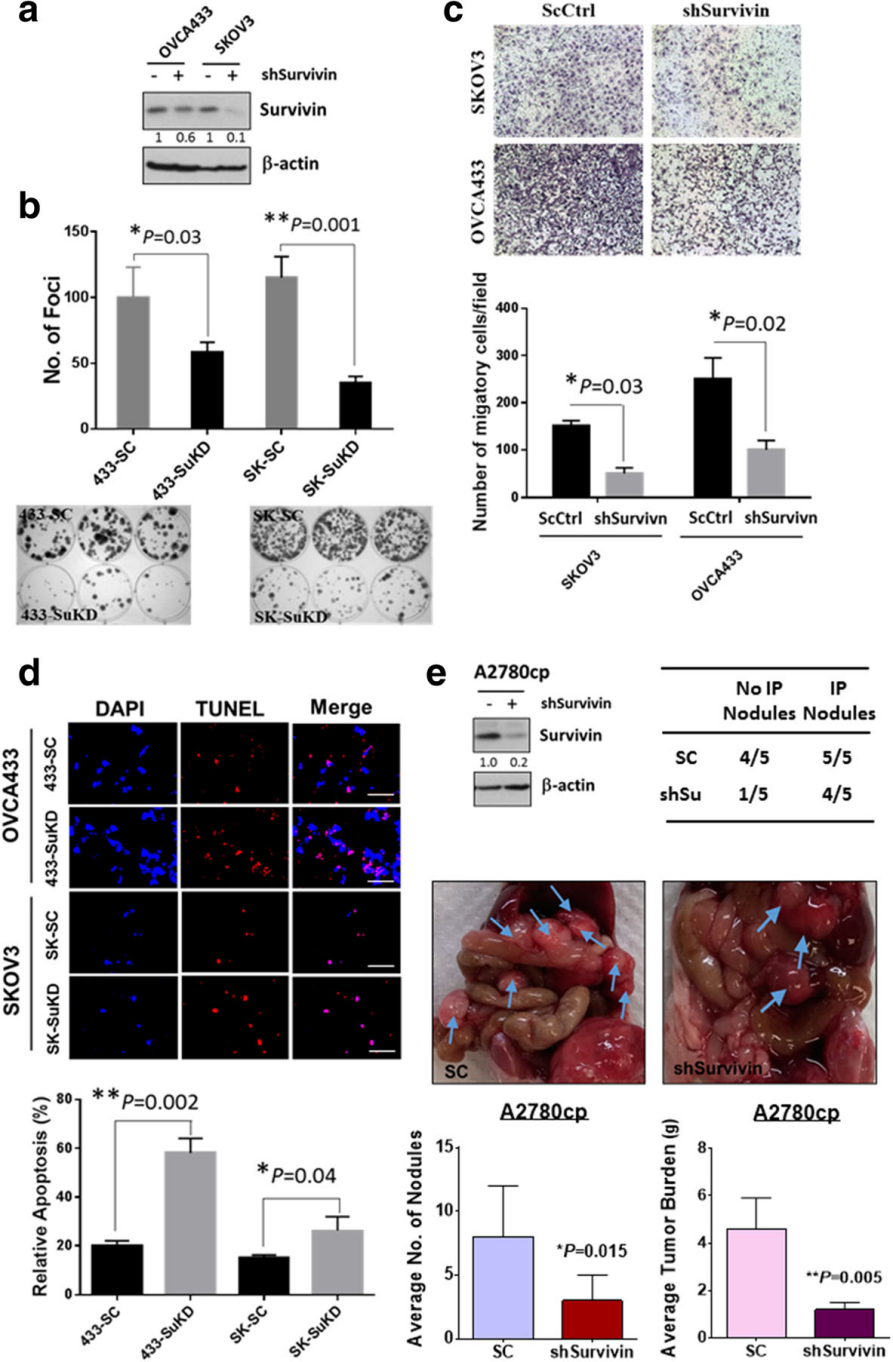
Survivin is required for anoikis resistance, cell proliferation, cell migration and tumor colonization of ovarian cancer cells. **a** Western blot analysis revealed that stable knockdown of survivin clones were established in OVCA433 and SKOV3 cells. **b** Focus formation assay demonstrated that knockdown of survivin significantly reduce both number and size of focus. **c** Stable knockdown of survivin also significantly impaired the cell migration capacity of ovarian cancer cell lines; SKOV3 and OVCA433 cells. **d** Upon anoikis assay treatment, results showed that depletion of survivin by shRNAi remarkably increase the number of TUNEL positive signals (RED signals) as compared with their scrambled controls (SC), indicating there is increased apoptotic rates of survivin-knockdown ovarian cancer cells. Bar, 100 μM. **e** Effects of survivin knockdown on ovarian cancer colonization. Western blot analysis confirmed 80% survivin was knockdown by lentiviral shRNAi approach. Both ovarian cancer cells with stably knockdown of survivin and scrambled control (SC) of A2780cp were i.p. injected into 5-weeks old female nude mice. Average number of tumor nodules and the tumor burden formed by survivin knockdown clone were much lower than the scrambled control and the tumor nodules stunning throughout the peritoneal cavity as photographed on Day 21 (3 weeks). The blue arrows point out the position of tumor nodules

The above findings showed that the miR-141/KLF12/survivin signaling cascade is involved in the enhancement of anoikis resistance in ovarian cancer cells. We questioned whether survivin has a role in modulating anoikis resistance in ovarian cancer cells, although mounting evidence has indicated that survivin is frequently overexpressed in human cancers [41] and involved in chemoresistance [42] and metastatic progression [43]. We first knocked down survivin using a shRNA approach in two ovarian cancer cell lines, OVCA433 and SKOV3 (Fig. 7a). Foci formation assays revealed that depletion of survivin reduced the number of foci by 48% to 65% in these two cell lines (Fig. 7b). In addition, the cell migratory capacity of survivin knockdown SKOV3 and OVCA433 clones was reduced by 2.5- to 3-fold compared with their scrambled controls (Fig. 7c). Moreover, upon treatment with the anoikis assay, the depletion of survivin in these two cell lines caused a substantial increase in the cell apoptotic rate by 2- to 3-fold (Fig. 7d).

Finally, to examine the functional role of survivin in metastatic potential of ovarian cancer cell growth and progression in peritoneal cavity, survivin knockdown was performed in another ovarian cancer cell line; A2780cp has been shown to highly express survivin and displays highly tumorigenicity in nude mice (Fig. 7e) [44]. The stable A2780cp survivin knockdown cells (80% knockdown) (Fig. 7e) (*N* = 5) and the vector control cells (*N* = 5) were intraperitoneally (i.p.) injected into nude mice, similar to the above in vivo tumorigenic assay, to examine the functional role of miR-141 in ovarian cancer cells. After 2 weeks, tumor growth was investigated by post-mortem examination. Compared with the scrambled control (SC), the survivin knockdown clone (shSu) resulted in fewer tumor nodules; tumor growth was observed in only one out of five mice in the survivin knockdown (shSu) group compared with four of five mice in the scrambled control (SC) group (Fig. 7e). Moreover, compared with the scrambled control group, knockdown of survivin resulted in a substantial reduction in the number of macroscopic tumor nodules in the omentum and the whole peritoneal cavity. The tumor mass of the survivin knockdown clone was ~4-fold less than that observed in the scrambled control (Fig. 7e). Taken together, these results suggest that survivin is required for anoikis resistance and metastatic progression of ovarian cancer cells in peritoneal metastases.

## Discussion

The acquisition of anoikis resistance in cancer cells in response to ECM detachment during cancer progression is responsible for the difficulties in treatment and unsatisfactory clinical management for different types of cancers. Thus far, the underlying molecular mechanisms of this process in cancer cells have received more attention recently, and many important findings have been reported [45, 46]. However, studies examining how ovarian cancer cells escape anoikis during metastasis still remain rare. In this study, we showed that the upregulation of miR-141 and the subsequent suppression of KLF12 are essential for anoikis resistance in ovarian cancer cells both in vitro and in vivo. We also demonstrated that the expression of survivin mRNA and protein increased drastically when miR-141 was expressed ectopically or Sp1 was overexpressed. The robust expression of survivin and its downstream target, XIAP, serves as a bridge between miR-141 and inhibition of intrinsic apoptotic pathway. Knocking down KLF12 produced results similar to miR-141-induced anoikis resistance. But depletion of survivin by shRNAi approach remarkably reduced anoikis resistance of ovarian cancer cells and suppressed their tumor colonization in peritoneal cavity of mice, suggesting survivin is the major downstream effector of miR-141/KLF12. Importantly, the inverse correlation between miR-141 and KLF12 expression was clinically confirmed of that the miR-141/KLF12/Sp1/survivin is a new signaling axis required for anoikis resistance of ovarian cancers during metastatic progression.

The dysregulation of miRNAs has been shown to be associated with cancer progression [47, 48]. The expression of the miR-200 family, including miR-141, is usually expressed differentially in various human cancers [49]. Previous studies have documented that the members of this family are downregulated and could suppresses epithelial-mesenchymal transition (EMT), while some members of this family are upregulated and could promote mesenchymal-epithelial transition (MET) in cancer development [50–52]. On the contrary, the miR-200 family in advanced and high-grade ovarian and breast cancers is frequently overexpressed and is closely related to distant metastasis [53, 54]. Indeed, the overexpressed miR-141 has been reported in association with increased chemoresistance and cell survival of ovarian cancer cells under oxidative stresses [55, 56]. In line with these studies, we showed that miR-141 is significantly upregulated in ovarian cancer cell lines and advanced metastatic ovarian cancers. Importantly, the expression of miR-141 showed an increased stepwise along the tumor stages, indicating miR-141 plays a crucial role in promoting ovarian cancer progression. Previous findings have shown that the miR-200 family is well-known for their EMT-suppressive and MET-promoting functions in other human cancers [57, 58]. However, little is known about their tumor-promoting abilities in metastatic ovarian cancers. In this study, a convergence of functional assays demonstrated that miR-141 not only elevates the viability of ovarian cancer cells in stressful microenvironments but also promotes anchorage independence, resulting in increased anoikis resistance in vitro and tumor growth and colonization in vivo. Anoikis resistance is the initial event and must be acquired by tumor cells when they undergo metastasis. In successful metastasis, anoikis resistance is important for cell survival in the circulation before the cells reach a distant location with a suitable ECM for reattachment to form a new niche. It is known that one miRNA could regulate a network of signaling through targeting a number of downstream targets [59]. In analysis across three well known miRNA target prediction programs; PicTar, miRanda and TargetScan 6.0, we found that there are 65 putative targets which can be further categorized into several main biological functions such as metabolic process, localization, cellular process, biological regulation, apoptotic process and cell survival. Intriguingly, some of the possible miR-141 targets are associated with cancer metastasis e.g. DLC1 [29], insulin receptor substrate 2 (IRS2) [30] and salt-inducible kinase 1 (SIK1) [31]. SIK1 is particularly of interest because SIK1 is able to interact with LKB1 which in turn, enhances p53-dependent anoikis and suppresses cancer metastasis [31]. However, our findings in this study showed that the overexpressed miR-141 could enhance anoikis resistance not only in p53-wildtype (OVCA433), but also in p53-mutated (A2780cp) and p53-deleted (SKOV3) ovarian cancer cell lines. That means there are other signalings governing anoikis resistance in ovarian cancers. Previous studies reported that some miR-141 targets function in cell survival and biological process could enhance chemoresistance and cell survival such as FUS and PAPP-A [27, 28]. These findings urged us to look into which possible targets in cell survival are involved in anoikis resistance of ovarian cancers. As expected, in combination of a series of functional and biochemical analyses, KLF12 is a direct target of miR-141 and importantly, the inverse relationship of KLF12 and miR-141 is significantly correlated with the advanced and metastatic ovarian cancers. KLF12 is a zinc finger protein that belongs to the relatively large SP/KLF family of transcription factors. The family consists of 25 members that regulate a wide range of cell functions, including different aspects of cancer progression. Most KLFs are upregulated in the early stages of cancer and are known to inhibit cancer cell invasiveness. However, the expression of EMT-suppressing KLFs is predominantly downregulated when the cells undergo systemic dissemination, which usually occurs in the advanced stages [60]. These findings support our clinicopathological analysis of the inverse correlation between miR-141 and KLF12 is related to cancer metastasis. Such inverse relationship between miR-141 and KLF12 is also confirmed in other human cancer types according to TCGA database. Furthermore, a recent study reported that KLF12 is a novel metastasis-suppressor gene and suppression of KLF12 resulted in anoikis resistance in lung cancer cells [61]. This suggests that miR-141 protects ovarian cancer cells from anoikis by suppressing KLF12 expression.

The study of anoikis is a new area in cancer research and thus, increasing evidence has suggested that the initiation and execution of anoikis is mediated by different pathways. The interplay between the intrinsic and extrinsic apoptotic pathways appears to be the most important. Although the recent study demonstrated KLF12 enhances anoikis resistance of lung metastatic cancer cells via regulating cell cycle progression [61], our findings in this study showed another mechanism of that the loss of KLF12 elevating the expression of survivin and its related intrinsic apoptotic pathway. Overexpression of survivin, which belongs to the inhibitor of apoptosis (IAP) proteins, may lead to cancer cell invasiveness, poor patient prognosis and low survival rates [62, 63]. The survivin anti-apoptotic network is mediated through the survivin-XIAP complex, which subsequently inhibits the activation of caspase-9 and the cleavage of caspase3 and PARP. Apart from being an apoptotic regulator, survivin also modulates cell cycle and proliferations [64]. Aberrant upregulation of survivin is frequently reported in many human cancers and involves in tumor growth, metastasis, chemoresistance and poor prognosis [64, 65]. Indeed, our in vitro and in vivo tumorigenic studies evidenced that survivin is required for anoikis resistance, tumor growth and cancer cell colonization in peritoneal metastases of ovarian cancer cells. This suggests that survivin is a crucial modulator of the intrinsic pathway in governing anoikis resistance of ovarian cancer cells.

Previous studies have suggested that the KLF family shares homology with the transcription factor Sp1, and therefore, they are grouped together as the Sp1/KLF family [39]. There are eight Sp1 binding sites with canonical or similar sequences [(G/T)(G/A)GGCG(G/T)(G/A)(G/A)(C/T)] in the survivin promoter, and thus, Sp1 is a key transcription factor for controlling the expression of survivin [40]. Therefore, we hypothesized that KLF12 and Sp1, which share homology, may compete for binding sites in the survivin promoter, inhibiting the transcriptional activation of survivin. As expected, our ChIP assay demonstrated that some Sp1 binding sites on survivin promoter are interfered by KLF12. Consistently, luciferase reporter assays confirmed this model by showing a significant increase in the luciferase signals under the control of the survivin promoter when Sp1 was co-expressed, while a significant suppression was observed when KLF12 was co-transfected with Sp1. This finding was further verified by western blot analyses of ovarian cancer cells transiently transfected with SP1 and KLF12 or with a stable Sp1 knockdown. Taken together, our study revealed that the overexpression of miR-141 augments anoikis resistance in ovarian cancer cells by targeting and repressing the expression of KLF12, which, in turn, competes for binding sites in the survivin promoter with Sp1. The subsequent increase in survivin then protects ovarian cancer cells against anoikis by blocking the intrinsic apoptotic activity.

## Conclusions

This study highlights a novel signaling cascade of miR-141/KLF12/Sp1/survivin in promoting anoikis resistance of ovarian metastatic cancer cells and suggests a unique molecular mechanism by which the intrinsic apoptotic pathway is impeded in ovarian cancer tumors by microRNA-mediated epigenetic regulation during ovarian cancer metastatic progression (Additional file 5: Figure S5). Conceivably, targeting this signaling axis could form the basis for a novel therapeutic strategy to treat or even prevent ovarian cancer micrometastases.

## Additional files

Additional file 1: Figure S3. (*Upper*) By using StarBase V2.0 (http://starbase.sysu.edu.cn/index.php) as analytic tool to analyze the inverse relationship between miR-141 and KLF12 in human cancers according Pan-Cancer miRNA-mRNA interaction maps from the Cancer Genome Atlas (TCGA) matched miRNA-Seq and RNA-Seq data, 11 out of 14 human cancer types showed the significant correlation. (*Lower*) The scatter chart showed the inverse correlation of miR-141 and KLF12 in 265 cases of ovarian serous cancers (Pearson correlation r = −0.15369, *P* value = 0.0122457). (TIF 327 kb)
Additional file 2: Figure S1. Enforced expression of miR141 increases anchorage-independent growth capacity of ovarian cancer cells. Soft agar assay showed that stably expression of miR141 in A2780cp (141-C2 and 141-C8) and SKOV3 (141-C2 and 141-C3) could significantly increase the number and size of the colonies as compared with their vector control (VC). The pictures showed the representative colony morphology and size of miR141 stable clones and vector control (VC). (TIF 311 kb)
Additional file 3: Figure S2. Putative targets for miR-141. Schematic presentation of the miR-141 seed matches in the human KLF12, E2F3 and MAP2K4 3'UTR introduced into the pmiRGLO luciferase 3'UTR constructs. Luciferase reporter assay to assess the interaction of miR-141 with the three putative target candidates, E2F3, MAP2K4 and KLF12 with the use of HEK293 cells. Co-transfection of miR-141 and KLF12 luciferase plasmids resulted in the strongest reduction of the luciferase reporter signals. (TIF 3533 kb)
Additional file 4: Figure S4. (*Upper*) Taking the promoter region (+/−1 k to TSS) of survivin: chr17:76209278–76211277, ten putative SEBs higher potential of Sp1 binding ability were shown. (*Lower*) The genomic sequences of primers targeting SBEs of Sp1 on the *survivin* promoter. (TIF 681 kb)
Additional file 5: Figure S5. A schematic diagram summaries the findings in this study. The overexpression of miR141 directly targets KLF12 expression. The reduced KLF12 expression allows Sp1 interacting with Survivin promoter that in turns, enhance the expression of survivin in both mRNA and protein levels. Through the stabilization of XIAP, the Survivin-XIAP complex which subsequently inhibiting the activation of caspase-9 and the cleavage of caspase-3 and PARP in the intrinsic apoptotic pathway and enhance anoikis resistance of advanced metastatic ovarian cancer cells in the ascetic tumor microenvironment. (TIF 320 kb)
