# Supplementary figures and images for: MicroRNA-141 enhances anoikis resistance in metastatic progression of ovarian cancer through targeting KLF12/Sp1/survivin axis
Source: Mol Cancer. 2017 Jan 17;16:11. doi: 10.1186/s12943-017-0582-2 (PMC5240442; doi:10.1186/s12943-017-0582-2)

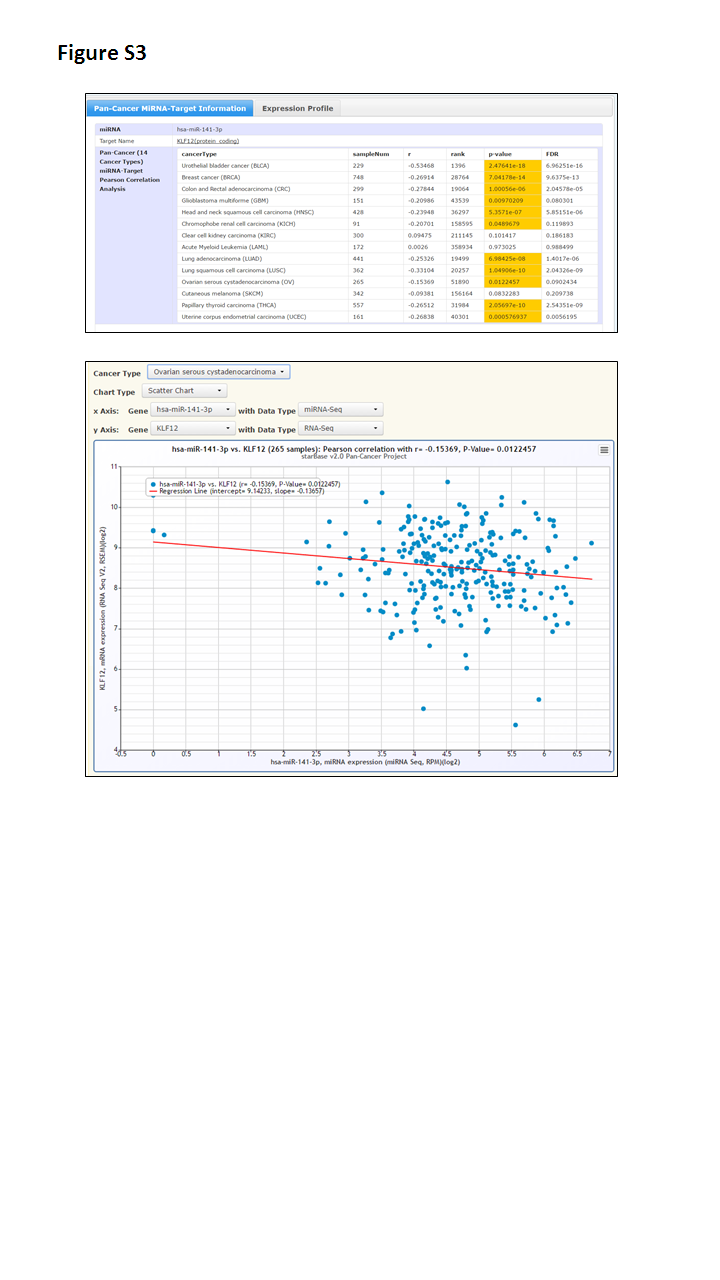

Supplement: Additional file 1: Figure S3. — (Upper) By using StarBase V2.0 (http://starbase.sysu.edu.cn/index.php) as analytic tool to analyze the inverse relationship between miR-141 and KLF12 in human cancers according Pan-Cancer miRNA-mRNA interaction maps from the Cancer Genome Atlas (TCGA) matched miRNA-Seq and RNA-Seq data, 11 out of 14 human cancer types showed the significant correlation. (Lower) The scatter chart showed the inverse correlation of miR-141 and KLF12 in 265 cases of ovarian serous cancers (Pearson correlation r = −0.15369, P value = 0.0122457). (TIF 327 kb) [file 12943_2017_582_MOESM1_ESM.tif]

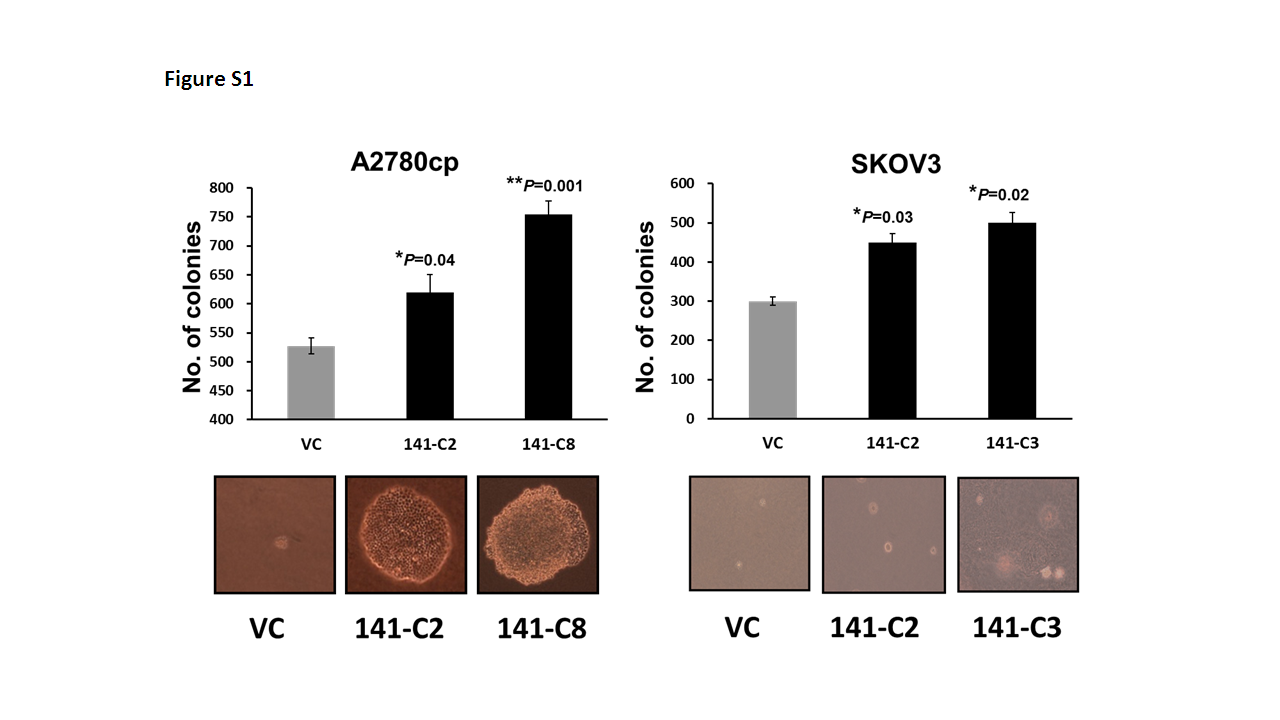

Supplement: Additional file 2: Figure S1. — Enforced expression of miR141 increases anchorage-independent growth capacity of ovarian cancer cells. Soft agar assay showed that stably expression of miR141 in A2780cp (141-C2 and 141-C8) and SKOV3 (141-C2 and 141-C3) could significantly increase the number and size of the colonies as compared with their vector control (VC). The pictures showed the representative colony morphology and size of miR141 stable clones and vector control (VC). (TIF 311 kb) [file 12943_2017_582_MOESM2_ESM.tif]

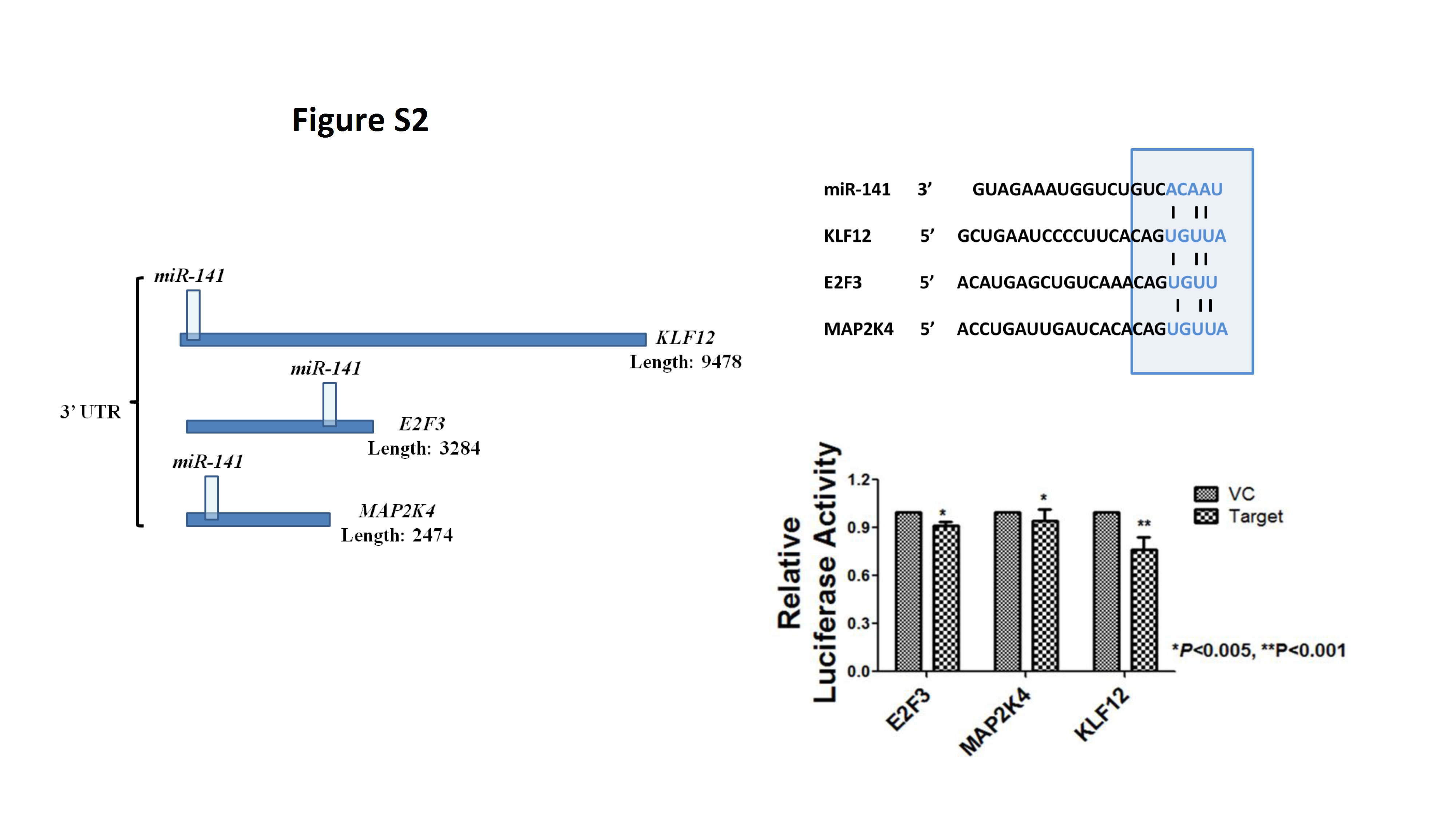

Supplement: Additional file 3: Figure S2. — Putative targets for miR-141. Schematic presentation of the miR-141 seed matches in the human KLF12, E2F3 and MAP2K4 3'UTR introduced into the pmiRGLO luciferase 3'UTR constructs. Luciferase reporter assay to assess the interaction of miR-141 with the three putative target candidates, E2F3, MAP2K4 and KLF12 with the use of HEK293 cells. Co-transfection of miR-141 and KLF12 luciferase plasmids resulted in the strongest reduction of the luciferase reporter signals. (TIF 3533 kb) [file 12943_2017_582_MOESM3_ESM.tif]

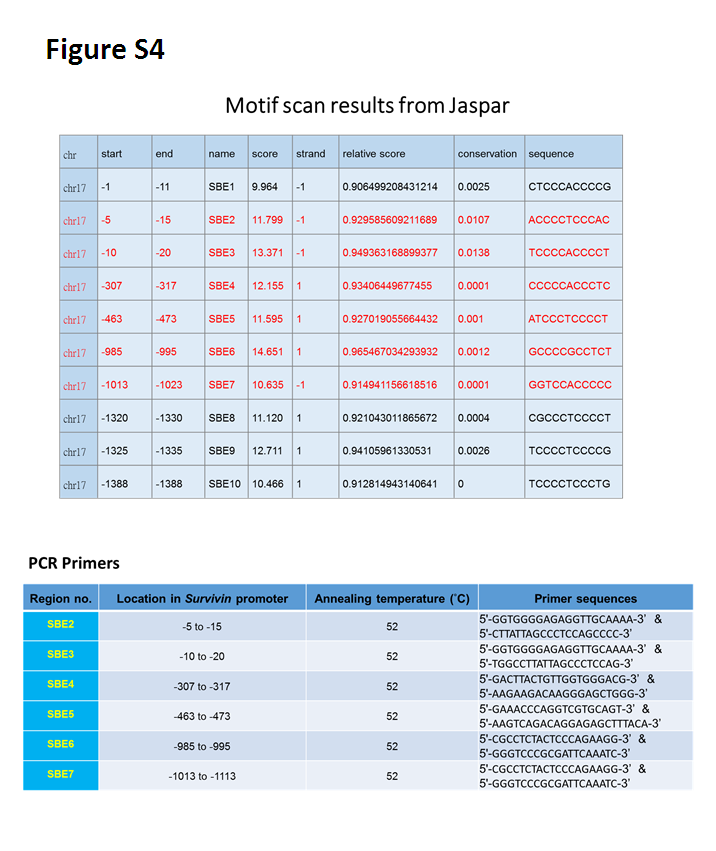

Supplement: Additional file 4: Figure S4. — (Upper) Taking the promoter region (+/−1 k to TSS) of survivin: chr17:76209278–76211277, ten putative SEBs higher potential of Sp1 binding ability were shown. (Lower) The genomic sequences of primers targeting SBEs of Sp1 on the survivin promoter. (TIF 681 kb) [file 12943_2017_582_MOESM4_ESM.tif]

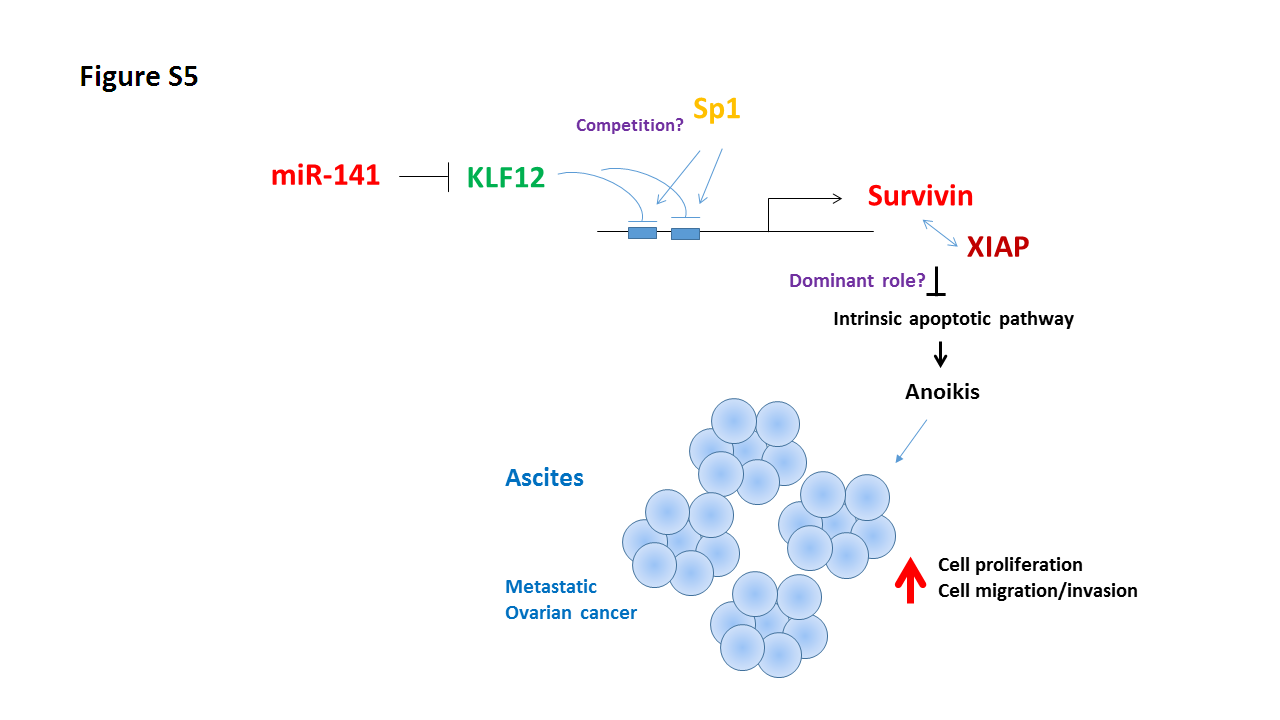

Supplement: Additional file 5: Figure S5. — A schematic diagram summaries the findings in this study. The overexpression of miR141 directly targets KLF12 expression. The reduced KLF12 expression allows Sp1 interacting with Survivin promoter that in turns, enhance the expression of survivin in both mRNA and protein levels. Through the stabilization of XIAP, the Survivin-XIAP complex which subsequently inhibiting the activation of caspase-9 and the cleavage of caspase-3 and PARP in the intrinsic apoptotic pathway and enhance anoikis resistance of advanced metastatic ovarian cancer cells in the ascetic tumor microenvironment. (TIF 320 kb) [file 12943_2017_582_MOESM5_ESM.tif]
